# Structural Investigations on Novel Non-Nucleoside Inhibitors of Human Norovirus Polymerase

**DOI:** 10.3390/v15010074

**Published:** 2022-12-27

**Authors:** Gilda Giancotti, Giulio Nannetti, Gilda Padalino, Martina Landini, Nanci Santos-Ferreira, Jana Van Dycke, Valentina Naccarato, Usheer Patel, Romano Silvestri, Johan Neyts, Roberto Gozalbo-Rovira, Jésus Rodríguez-Díaz, Joana Rocha-Pereira, Andrea Brancale, Salvatore Ferla, Marcella Bassetto

**Affiliations:** 1 School of Pharmacy and Pharmaceutical Sciences, Cardiff University, Cardiff CF10 3NB, UK; 2 Medical School, Faculty of Medicine, Health and Life Science, Swansea University, Swansea SA2 8PP, UK; 3 KU Leuven—Rega Institute, Department of Microbiology, Immunology and Transplantation, 3000 Leuven, Belgium; 4 Department of Drug Chemistry and Technologies, Sapienza University of Rome, Piazzale Aldo Moro 5, 00185 Rome, Italy; 5 Department of Microbiology, School of Medicine, University of Valencia, 46010 Valencia, Spain; 6 Vysoká Škola Chemiko-Technologiká v Praze, 165 00 Prague, Czech Republic; 7 Department of Chemistry, College of Science and Engineering, Swansea University, Swansea SA2 8PP, UK

**Keywords:** human norovirus, RdRp inhibitors, computer-aided drug design

## Abstract

Human norovirus is the first cause of foodborne disease worldwide, leading to extensive outbreaks of acute gastroenteritis, and causing around 200,000 children to die annually in developing countries. No specific vaccines or antiviral agents are currently available, with therapeutic options limited to supportive care to prevent dehydration. The infection can become severe and lead to life-threatening complications in young children, the elderly and immunocompromised individuals, leading to a clear need for antiviral agents, to be used as treatments and as prophylactic measures in case of outbreaks. Due to the key role played by the viral RNA-dependent RNA polymerase (RdRp) in the virus life cycle, this enzyme is a promising target for antiviral drug discovery. In previous studies, following in silico investigations, we identified different small-molecule inhibitors of this enzyme. In this study, we rationally modified five identified scaffolds, to further explore structure–activity relationships, and to enhance binding to the RdRp. The newly designed compounds were synthesized according to multiple-step synthetic routes and evaluated for their inhibition of the enzyme in vitro. New inhibitors with low micromolar inhibitory activity of the RdRp were identified, which provide a promising basis for further hit-to-lead optimization.

## 1. Introduction

Human norovirus is one of the leading causes of acute gastroenteritis worldwide, responsible for approximately 18% of all cases of diarrhea and vomit [1,2], with this value increasing to almost 58% when considering only outbreaks caused by contaminated food [3]. Human norovirus infections are associated with significant economic losses and societal costs every year [4]. Currently, a vaccine is still unavailable, while the development of antiviral agents is limited, mainly due to the high genetic variability and rapid evolution of norovirus strains, and a lack of animal models and robust cell culture systems [5]. While four candidate vaccines are now in phase I or phase II clinical trials [6], most antiviral agents for treating norovirus infections are currently investigated at early stages of preclinical studies, with the exception of CMX521, a nucleoside analogue in phase I [7], and nitazoxanide, an FDA-approved antiparasitic agent with broad-spectrum antiviral activity, which completed phase II trials [8].

**Figure 1:**
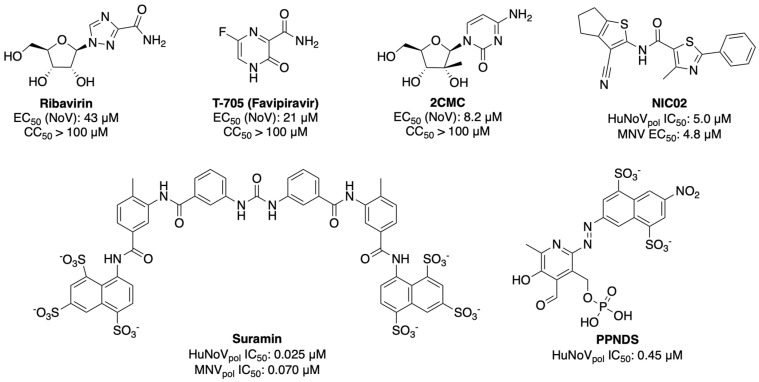
Chemical structures of previously reported inhibitors of norovirus RdRp [16].

Noroviruses belong to the *Caliciviridae* family, with a positive-sense, single-stranded RNA genome, and are classified into 10 genogroups (GI-GX) and 48 genotypes [9]. Most large outbreaks of gastroenteritis are caused by GII.4 strains [10], and by GI sub-variants [11,12]. Norovirus genome is characterized by three open reading frames (ORF1–3). When translated, ORF1 produces a polyprotein precursor, which is cleaved by a virus-encoded 3C-like cysteine protease (3CLpro) into seven non-structural proteins (NS1–7), all with essential roles in viral replication [13,14]. The RNA-dependent RNA polymerase (RdRp) NS7 represents a particularly promising antiviral target, due to its pivotal role in transcription and replication of the viral genome, and to the proven success of polymerase and reverse transcriptase inhibitors in antiviral drug discovery [15]. A range of inhibitors of norovirus RdRp has been reported, including both nucleoside and non-nucleoside agents, as summarized in Figure 1 [16].

**Figure 2:**
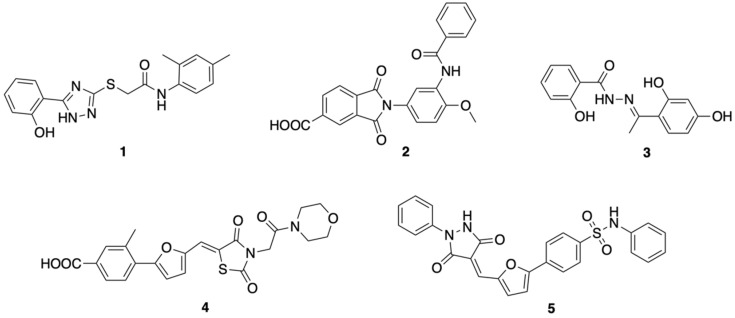
Micromolar inhibitors of norovirus RdRp previously identified by our group through a docking-based virtual screening approach [18].

Several crystal structures of human and murine norovirus (MNV) RdRps have been determined, also in complex with different inhibitors [17]. Starting from the in silico analysis of some of these structures (PDB IDs 3UR0 and 4LQ3), we previously performed a combined structure- and ligand-based virtual screening of commercial libraries of drug-like compounds, which yielded multiple in vitro inhibitors of norovirus RdRp, exhibiting micromolar activities [18]. Their chemical structures are shown in Figure 2.

We recently reported initial structure–activity relationships and optimization studies for scaffolds 4 and 5, which enabled the identification of low- and sub-micromolar inhibitors of human norovirus replication in cell-based assays [19,20]. We here discuss the preparation of the first series of novel analogues of compounds **1–3**, to develop structure–activity relationships of these different chemical scaffolds for the inhibition of norovirus RdRp. In addition, we report the exploration of further structural modifications for all scaffolds **1–5**, introducing polar or negatively charged functional groups, which could enhance binding to the viral RdRp. The newly designed compounds were synthesized and evaluated for their ability to inhibit human norovirus RdRp in vitro, and to interfere with the replication of MNV in cell-based assays.

## 2. Materials and Methods

### 2.1. Molecular Modelling Studies

All molecular modelling experiments were performed on Asus WS X299 PRO Intel^®^ i9-10980XE CPU @ 3.00 GHz × 36 running Ubuntu 18.04 (graphic card: GeForce RTX 2080 Ti). Molecular Operating Environment (MOE) 2019.10 [21] and Maestro (Schrödinger Release 2020-2) [22] were used as molecular modelling software. The structures of the newly designed compounds were built in MOE, saved in .sdf format and prepared using the Maestro LigPrep tool by energy minimizing the structures (OPLS_2005 force filed), generating possible ionization states at pH 7 ± 2, tautomers, all possible stereoisomers per ligand and low-energy ring conformers. The RdRp structure in complex with PPNDS was downloaded from the PDB data bank (http://www.rcsb.org/ (accessed on 9 October 2022); PDB code 4LQ3). The protein was pre-processed using the Schrödinger Protein Preparation Wizard by assigning bond orders, adding hydrogens and performing a restrained energy minimization of the added hydrogens using the OPLS_2005 force field. An 11 Å docking grid (inner-box 10 Å and outer-box 21 Å) was prepared using the co-crystallized PPNDS as the centroid. Molecular docking studies were performed using Glide SP precision keeping the default parameters and setting 5 as the number of output poses per input ligand. The output poses were saved as mol2 files. The docking results were visually inspected for their ability to bind the active site.

### 2.2. Synthetic Chemistry

All solvents and reagents were used as obtained from commercial sources unless otherwise indicated. All solvents used for chromatography were HPLC grade from Fisher Scientific (UK). All reactions were performed under a nitrogen atmosphere; ^1^H, ^13^C, ^19^F and ^31^P-NMR spectra were recorded with a Bruker Avance III HD spectrometer operating at 500 MHz for ^1^H and 125 MHz for ^13^C, with Me_4_Si as internal standard. Deuterated chloroform or dimethylsulfoxide (DMSO) were used as the solvents for NMR experiments, unless otherwise stated; ^1^H chemical shifts values (δ) are referenced to the residual non-deuterated components of the NMR solvents (δ = 7.26 ppm for CHCl_3_, etc.). The ^13^C chemical shifts (δ) are referenced to CDCl_3_ (central peak, δ = 77.0 ppm). TLC was performed on silica gel 60 F254 plastic sheets. Flash column chromatography was performed using a Biotage Isolera One automated system. UPLC-MS analysis was conducted on a Waters UPLC system with both Diode Array detection and Electrospray (+′ve and −′ve ion) MS detection. The stationary phase was a Waters Acquity UPLC BEH C18 1.7 µm 2.1 50 mm column. The mobile phase was LC-MS-grade H_2_O containing 0.1% formic acid (A) and LC-MS grade MeCN containing 0.1% formic acid (B). Column temperature: 40 °C. Sample diluent: MeCN. Sample concentration: 1 μg/mL. Injection volume: 2 μL. Three alternative methods were used: Linear gradient standard method (A): 90% A (0.1 min), 90–0% A (2.5 min), 0% A (0.3 min), 90% A (0.1 min); flow rate 0.5 mL/min. Linear gradient standard method (B): 90% A (0.1 min), 90–0% A (2.1 min), 0% A (0.8 min), 90% A (0.1 min); flow rate 0.5 mL/min. Linear gradient standard method (C): 90% A (0.1 min), 90–0% A (1.5 min), 0% A (1.4 min), 90% A (0.1 min); flow rate 0.5 mL/min. High resolution mass spectra (HRMS) were measured in positive mode electrospray ionization (ES+). All compounds tested in biological assays were >95% pure. The purity of intermediates that were not biologically evaluated was >90%, unless otherwise stated. Preparation and characterization of all intermediates are fully described in the Supplementary Information, along with details for the preparation of the final target products **1**, **1a**–**f**, **2a**–**h**, **3a**–**f**, **4a**–**c** and **5a**.

### 2.3. Norovirus Polymerase Assays

#### 2.3.1. Compounds

All compounds were dissolved in an appropriate volume of DMSO to achieve stock solutions of 25 mM, 50 mM or 100 mM and stored in aliquots at −20 °C. Compounds were tested in a preliminary fluorescent RdRp activity assay at 20 μM in triplicates. Dose-response curves were obtained testing inhibitors at 0.1 μM, 1 μM, 10 μM, 25 μM, 50 μM, and 100 μM. Each compound concentration was tested at least in duplicate. In the gel-based assay, compounds were tested at 100 μM.

#### 2.3.2. Recombinant RdRp Expression and Purification

Recombinant norovirus RdRp GII.4 Groningen 2014 (GenBank accession number CRL46960.1) was expressed and purified as previously described [23].

#### 2.3.3. Quantitative Fluorescent RdRp Activity

Norovirus RdRp activity was quantified by monitoring the formation of double-stranded RNA (dsRNA) from a single stranded homopolymeric template, poly(C) (Sigma Aldrich, Burlington, MA, USA) using PicoGreen fluorescent dye (Thermo Fisher Scientific, Waltham, MA, USA). RdRp assays were performed in 96-well plates, and each reaction mixture contained 200 ng enzyme, 0.23 mM rGTP (Promega UK), 250 ng poly(C) RNA, 2.5 mM MnCl_2_, 5 mM dithiothreitol (DTT), 0.01% bovine serum albumin (BSA), and 0.005% Tween 20 in 20 mM Tris-HCl (pH 7.5), with a final reaction mixture of 50 μL. The known norovirus RdRp inhibitor PPNDS was used as a positive control in each experiment. RdRp was incubated for 10 min at room temperature in the presence of the test compounds or the compound vehicle DMSO (0.5% vol/vol) before addition into the reaction mixture. The reaction was allowed to run 10 min at 30 °C in a thermocycler (Eppendorf, Hamburg, Germany), then was terminated with 5 mM EDTA. Successively, reaction mixtures were transferred into black 96-well microplates (Sigma Aldrich, Burlington, MA, USA), and 165 μL PicoGreen (1:680 [vol/vol] diluted in TE buffer, pH 7.5) was added. The mixture was covered from light, incubated for 5 min, and the fluorescence intensity was measured using a CLARIOstar plate reader (BMG Labtech, Ortenberg, Germany) at excitation and emission wavelengths of 485 nm and 520 nm, respectively. A separate control reaction was used to quantify and remove background fluorescence. GraphPad Prism version 8.0 (La Jolla, CA, USA) was used to plot the IC_50_ values, applying a nonlinear regression-curve fit (inhibitor concentration versus normalized response with variable slope equation). IC_50_ corresponded to the compound concentration that inhibited by 50% the norovirus RdRp activity.

#### 2.3.4. Gel-Based Assay

Compounds inhibition of the RdRp primed elongation activity was examined in a gel-based assay using the RNA template (PE44-NoV). Reactions were performed using 1 μM PE44-NoV. Each reaction mixture contained 400 ng RdRp, 0.4 mM rGTP (Promega UK), 5mM dithiothreitol (DTT), 2.5 mM MnCl_2_, 1 unit Ribolock RNAse inhibitor (Thermo Fisher Scientific), and 20 mM Tris-HCl (pH 7.5) in a 25 μL final volume. RdRp was incubated for 10 min at room temperature in the presence of the test compounds or the compound vehicle DMSO (0.5% vol/vol) before addition into the reaction mixture. The known norovirus RdRp inhibitor PPNDS was used as a positive control in each experiment. The reaction mixture was incubated for 6 h at 30 °C in a thermocycler. After addition of denaturating sample buffer, PE44-NoV products were run on Biorad Mini Protean 15% TBE Urea denaturating gels for 95 min at 100V. Gels were stained with SYBR Green II (Invitrogen, Waltham, MA, USA) diluted 1:5000 [vol/vol] in TBE buffer and visualized on a GelDoc imager (Bio-Rad, Hercules, CA, USA).

### 2.4. Antiviral and Cytotoxicity Assays

The antiviral and cytotoxicity effects of selected compounds were evaluated in a MNV cytopathic effect (CPE) reduction assay, according to a previously described protocol [24].

#### 2.4.1. Cells and Virus

MNV (virus strain MNV-1.CW1) was propagated in RAW 264.7 cells grown in DMEM (Life Technologies, Gent, Belgium) supplemented with 10% or 2% fetal bovine serum (FBS), 2 mM L-glutamine, 20 mM HEPES, 0.075 g/L sodium bicarbonate, 1 mM sodium pyruvate, 100 U/mL penicillin and 100 g/mL streptomycin at 37 °C in a humidified atmosphere of 5% CO_2_.

#### 2.4.2. MNV Antiviral Assay

The antiviral activity of the compounds was determined using an MTS [3-(4,5-dimethylthiazol-2-yl)-5-(3-carboxymethoxyphenyl)-2-(4-sulfophenyl)-2H-tetrazolium]-based cytopathic effect (CPE) reduction assay. RAW 264.7 cells (1 × 10^4^ cells/well) were seeded in a 96-well plate and infected with MNV [at the multiplicity of infection (MOI) of 0.001] in the presence (or absence) of a dilution series of compounds. Infected cells were incubated for 3 days at 37 °C, i.e., until complete CPE was observed in infected untreated cells. Then, an MTS/phenazinemethosulfate (MTS/PMS) stock solution [containing 2 mg/mL MTS (Promega, Leiden, The Netherlands) and 46 g/mL PMS (Sigma–Aldrich, Bornem, Belgium) in PBS at pH 6–6.5] was diluted 1:20 in MEM (Life Technologies, Gent, Belgium) and 75 μL were added to each well. After 2 h of incubation at 37 °C, the optical density (OD) was read at 498 nm. The %CPE reduction was calculated as [(OD_treated_)_MNV_ − OD_VC_]/[OD_CC_ − OD_VC_] × 100, where OD_CC_ represents the OD of the uninfected untreated cells, whereas OD_VC_ and (OD_treated_)_MNV_ represent the OD of infected untreated cells and virus-infected cells treated with a compound concentration, respectively. The 50% effective concentration (EC_50_) was defined as the compound concentration that protected 50% of the cells from virus-induced CPE [24].

#### 2.4.3. Cytotoxicity

The cytotoxicity of the compounds was evaluated by the MTS method [24], by incubating uninfected cells to the same concentrations of compounds tested in the antiviral assay for 3 days. The %cell viability was calculated as (OD_treated_/OD_CC_) × 100, where OD_CC_ is the OD of uninfected untreated cells and OD_treated_ are uninfected cells treated with compound. The CC_50_ was defined as the compound concentration that reduces the cell viability by 50% [24].

## 3. Results and Discussion

### 3.1. Rational Design of Novel Analogues and Molecular Docking Studies

**Figure 3:**
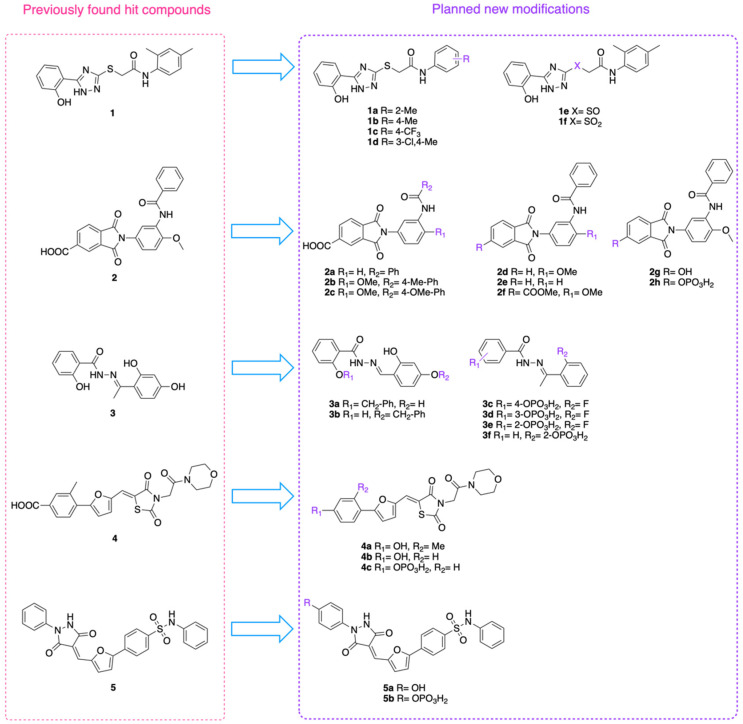
Summary of the planned structural modifications to the scaffolds of hit compounds **1**–**5**.

A first series of compounds was designed to explore the importance of the main structural features of hit molecules **1**–**2** (**1a**–**e**, **2a**–**f**), to evaluate structure–activity relationships for their inhibitory effect of norovirus RdRp. The potential to expand the scaffold of **3** was also explored with the design of analogues **3a**,**b**, while for all hits **1**–**5**, the introduction of polar or negatively charged functional groups was envisaged in compounds **1f**,**g**, **2h**,**i**, **3c**–**f**, **4a**–**c** and **5a**,**b**. This modification was designed to mimic the phosphate and phenolic OH groups of PPNDS, and of the RNA natural ligand, to potentially enhance the compounds binding affinity to the viral RdRp. Phenolic hydroxy, sulphone and phosphate groups were introduced in the structures of the original hits **1**–**5** (Figure 3).

**Figure 4:**
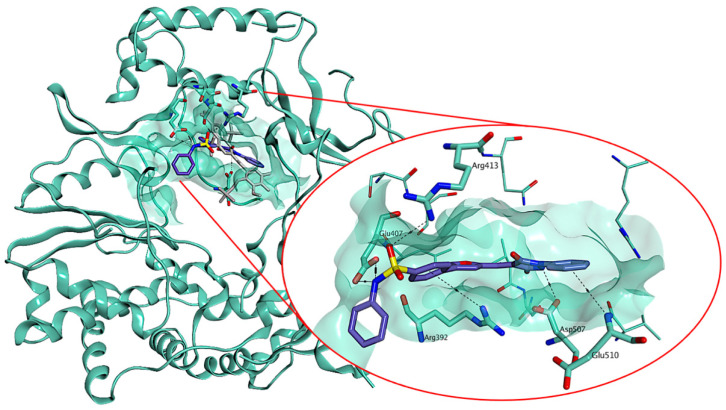
Previously proposed binding mode for **5** (carbon atoms in purple) to the human norovirus RdRp, overlapped with the co-crystallized ligand PPNDS (atoms in grey) [18,19]. The binding site area is represented as green molecular surface. Human norovirus RdRp is represented as green ribbon, with carbon atoms in green. Black dashed lines represent non-bonded interactions (e.g., hydrogen bonds, electrostatic interactions) between the ligand and the amino acid residues of the protein.

Prior to their synthesis, the predicted binding modes to HuNoV RdRp main binding site was evaluated for the newly designed compounds **2g**,**h**, **3a**–**f**, **4a**–**c** and **5a**,**b**. The best previously identified hit, **5** (IC_50_ 5.6 µM), had been evaluated with competitive binding studies with PPNDS [18,19], which suggested it likely occupies the central portion of the nucleic acid binding cleft of the RdRp (Figure 4), in a similar binding region to that of PPNDS. The phenyl-benzenesulfonamide portion of **5** is predicted to interact through its sulfonamide group with Glu407 and Arg413, and through its central benzene ring with Arg392. The phenyl-pyrazolidine portion of **5** is predicted to make non-bonded interactions with Asp507 and Glu510.

**Figure 5:**
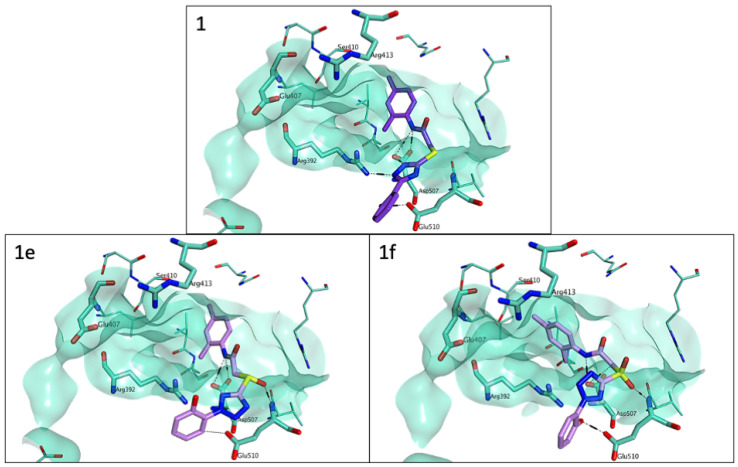
Proposed binding mode for **1** (carbon atoms in purple) and **1e**,**f** (carbon atoms in lilac) to the human norovirus RdRp (PDB ID 4LQ3). The binding site area is represented as green molecular surface. Human norovirus RdRp is represented as green ribbon, with carbon atoms in green. Black dashed lines represent non-bonded interactions (e.g., hydrogen bonds, electrostatic interactions) between the ligand and the amino acid residues of the protein.

**Figure 6:**
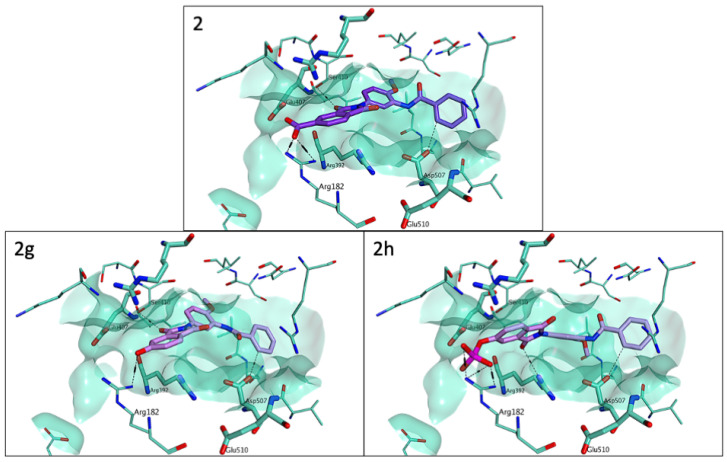
Proposed binding mode for **2** (carbon atoms in purple) and **2g**,**h** (carbon atoms in lilac) to the human norovirus RdRp (PDB ID 4LQ3). The binding site area is represented as green molecular surface. Human norovirus RdRp is represented as green ribbon, with carbon atoms in green. Black dashed lines represent non-bonded interactions (e.g., hydrogen bonds, electrostatic interactions) between the ligand and the amino acid residues of the protein.

**Figure 7:**
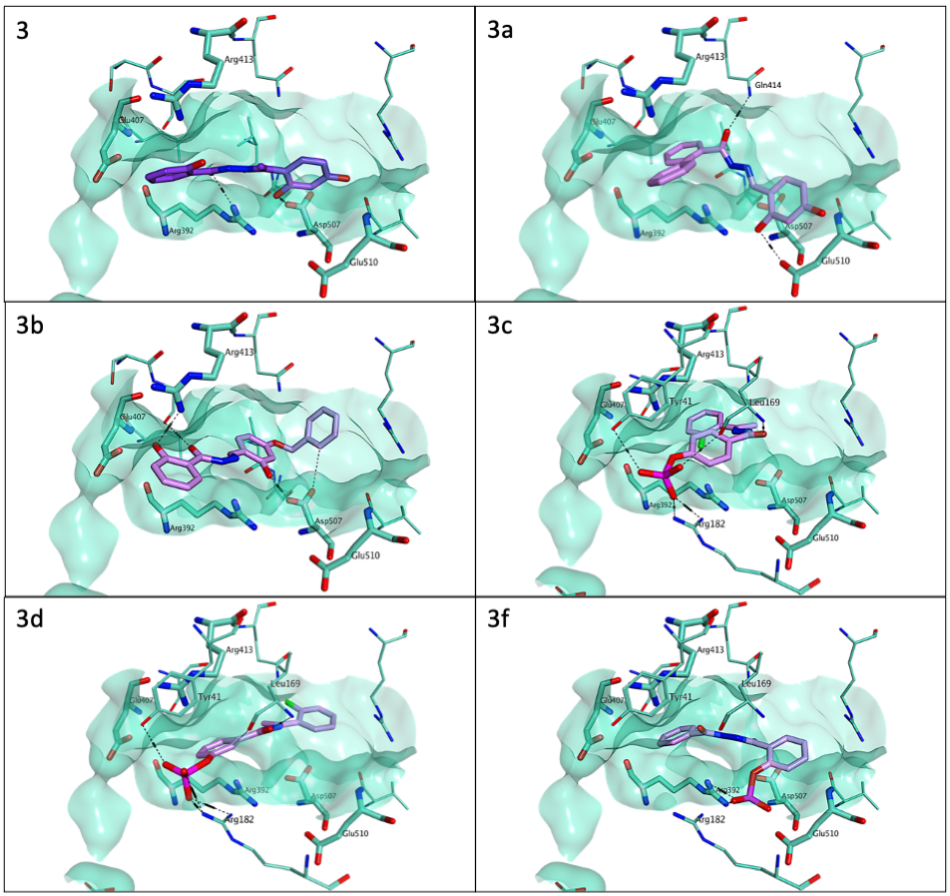
Proposed binding mode for **3** (carbon atoms in purple), **3a**–**d** and **3f** (carbon atoms in lilac) to the human norovirus RdRp (PDB ID 4LQ3). The binding site area is represented as green molecular surface. Human norovirus RdRp is represented as green ribbon, with carbon atoms in green. Black dashed lines represent non-bonded interactions (e.g., hydrogen bonds, electrostatic interactions) between the ligand and the amino acid residues of the protein.

**Figure 8:**
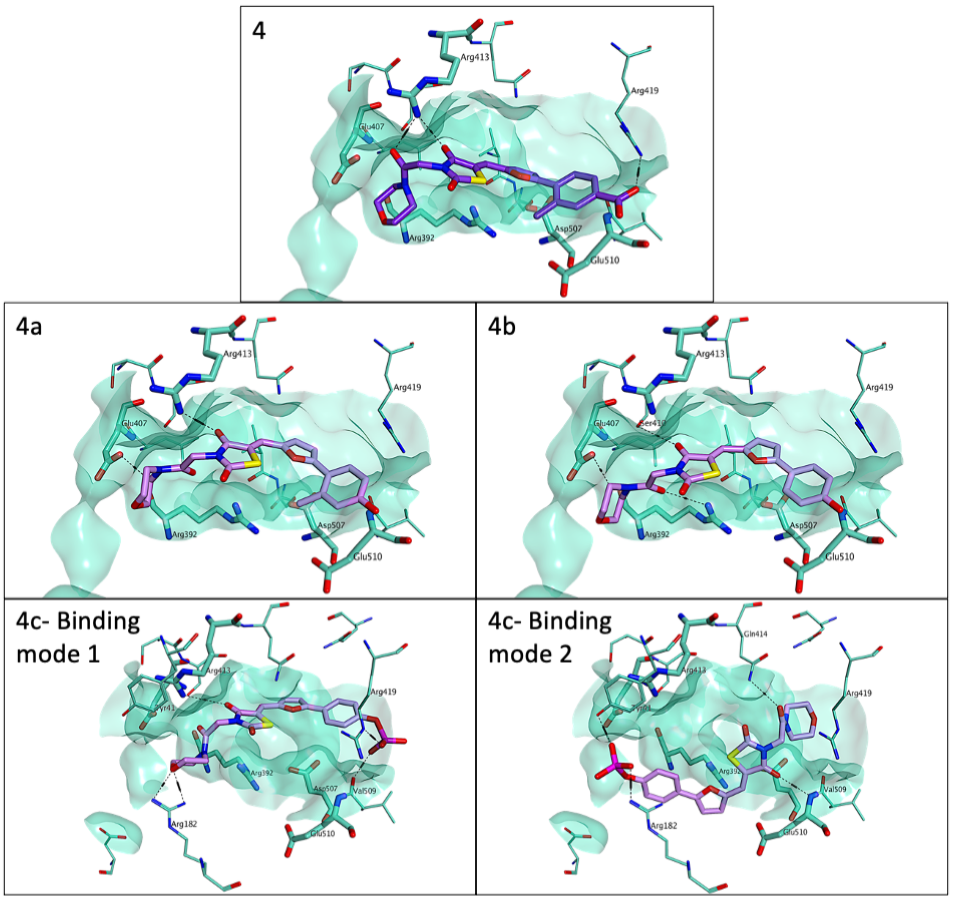
Proposed binding mode for **4** (carbon atoms in purple) and **4a**–**c** (carbon atoms in lilac) to the human norovirus RdRp (PDB ID 4LQ3). The binding site area is represented as green molecular surface. Human norovirus RdRp is represented as green ribbon, with carbon atoms in green. Black dashed lines represent non-bonded interactions (e.g., hydrogen bonds, electrostatic interactions) between the ligand and the amino acid residues of the protein.

**Figure 9:**
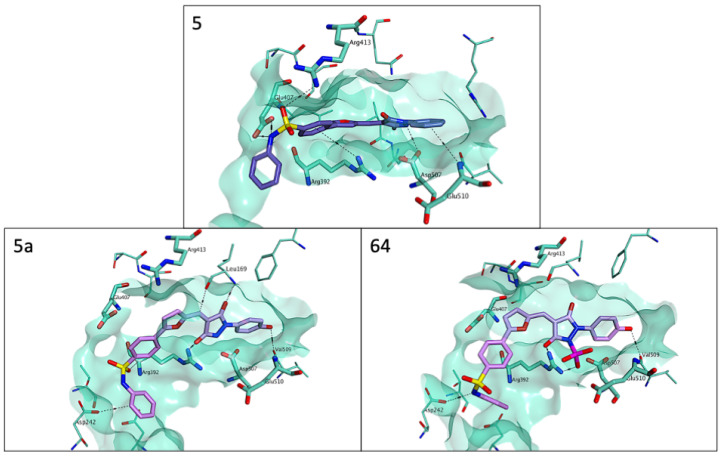
Proposed binding mode for **5** (carbon atoms in purple), **5a** and **64** (carbon atoms in lilac) to the human norovirus RdRp (PDB ID 4LQ3). The binding site area is represented as green molecular surface. Human norovirus RdRp is represented as green ribbon, with carbon atoms in green. Black dashed lines represent non-bonded interactions (e.g., hydrogen bonds, electrostatic interactions) between the ligand and the amino acid residues of the protein.

Molecular docking results obtained for the newly designed derivatives on the binding site of **5** and PPNDS, using Glide SP [22], are summarized in Figure 5, Figure 6, Figure 7, Figure 8 and Figure 9.

As shown in Figure 5, original hit **1** is predicted to occupy a slightly different subportion of the main binding site of NoV RdRp compared with **5**, in an extended conformation, forming direct contacts with Arg392, Asp507, and Glu510. While analogues **1a**–**d** were designed to test the importance of the two methyl substituents on the second phenyl ring of the scaffold, oxidation of the sulfur atom to sulfoxide (**1e**) appears to induce a significant conformational change to the scaffold, with loss of the direct hydrogen bonds with Arg392 and Asp507, and formation of an additional interaction with the backbone amide nitrogen of Asp507. The full oxidation of the sulfide to sulfone (**1f**) is associated with the loss of the hydrogen-bond opportunity with Arg392, and restoration of the hydrogen bond with the sidechain of Asp507.

Figure 6 summarizes the predicted binding of the scaffold of **2** to HuNoV RdRp.

Compound **2** is predicted to occupy the same central portion of the site observed for **5**, with fewer interactions. An electrostatic interaction is observed with the sidechain of Arg182, along with a hydrogen bond with the sidechain of Ser410, and an additional non-bonded interaction with the sidechain of Asp507. Analogues **2a**–**f** were designed to explore the importance of the methoxy substituent on the central ring (**2a**,**e**), the potential to insert substituents in the terminal phenyl ring (**2b**,**c**), the role of the carboxylate group (**2d**) and of its negative charge at physiological pH (**2f**). Replacement of the carboxylic acid with a hydroxy group (**2g**) was envisaged instead to enable the introduction a phosphate functionality in the scaffold (**2h**). While the predicted binding of **2g** is associated with retention of the key interactions observed for **2**, **2h** appears to enable an improved binding, with the formation of stronger interactions with the sidechain of Arg182, and with a new hydrogen bond with the sidechain of Arg392.

The predicted binding to the HuNoV RdRp observed for **3** and its analogues is summarized in Figure 7.

Compound **3** has a smaller molecular scaffold compared with all the other previous hits, leading to a smaller number of predicted interactions. Compound **3** appears to occupy the central part of this large pocket, making two hydrogen bonds, with the sidechains of Arg392 and Asp507. New analogues **3a**,**b** were designed to increase the spatial occupation of the target pocket for this scaffold. Compound **3a** is associated with a different binding orientation compared with **3**, shifted towards the right hand-side of the pocket, making a different set of interactions involving the same functional groups observed for **3**, and the sidechains of Gln414 and Glu510. A successful molecular extension appears instead to be achieved with **3b**, which maintains the same orientation of **3**, but enables the occupation of an additional portion of space, and an increased number of interactions with Arg413, Ser410 and Asp507. The four phosphate analogues **3c**–**f** were designed to increase the interactions between the scaffold and the enzyme. Insertion of a phosphate group at the *para* (**3c**) or *meta* (**3d**) position of the hydrazide ring is associated with strong predicted interactions with the sidechains of Arg182 and Tyr41, and the backbone groups of Leu169. A phosphate substituent at the *ortho* position of this ring (**3e**) is not associated with an enhanced binding potential, while insertion of this group at the *ortho* position of the hydrazone ring (**3f**) leads to a predicted strong interaction with the sidechain of Arg392, although the overall binding of the scaffold is perturbed in comparison to **3**.

As shown in Figure 8, **4** is predicted to occupy the central portion of the main binding site of the enzyme in an extended conformation, with an electrostatic interaction with Arg419, and a double interaction with Arg413.

For both analogues where the carboxylate group has been replaced by an OH (**4a**,**b**), a similar binding mode to that of the parent compound is expected, with the predicted loss of the direct contact with Arg419, but conferring the potential for additional contacts with the sidechains of Arg392, Glu407 and Ser410. Replacement of the original carboxylate with a phosphate group (**4c**) is associated with an overall enhancement of direct contact opportunities. In the docking analysis, two different binding modes were observed for **4c**: one with the same orientation of **4**, presenting an increased number of direct contacts with Arg182, Arg419 and Val509, and a second one, recurring with a comparable frequency in the best binding poses obtained, with a flipped orientation. In this case, an increased number of contacts is observed with the sidechains of Tyr41, Arg182 and Gln414, and the backbone NH group of Glu510.

Finally, Figure 9 summarizes the predicted binding for the new analogues of **5**.

Insertion of a phenolic OH group in the terminal ring of **5** (**5a**) is associated with a retained occupation of the binding site, although the contact pattern is different and it mainly involves the backbone groups of Leu169 and Val509, and the sidechains of Asp242 and Arg392. Interestingly, no binding poses could be obtained for the phosphate derivative **5b**, likely due to an excessive molecular length for this analogue, whose chemical structure is rigid. As detailed in Section 3.2 below, the synthesis of **5b** could not be achieved, as the main phosphorylation site on **5a** was the heterocyclic nitrogen, observed in the undesired product of the final reaction to make **5a**, compound **64**. The unplanned **64** is predicted to make different direct contacts at the level of the RdRp main binding site, mainly with the sidechains of Asp242 and Arg393, and the backbone carbonyl of Val509.

### 3.2. Synthesis of the Newly Designed Compounds

**Scheme 1:**
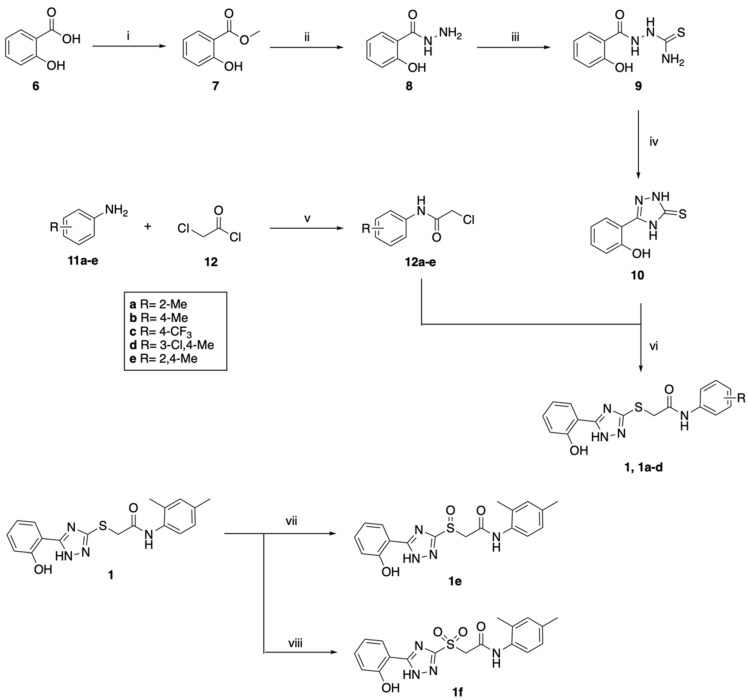
Preparation of **1** and its novel target analogues **1a**–**f**. *Reagents and conditions*: (i) H_2_SO_4_, MeOH, reflux, o.n. (88%); (ii) NH_2_NH_2_*H_2_O, EtOH, reflux, o.n. (94%); (iii) NH_4_SCN, EtOH, HCl, reflux, o.n. (58%); (iv) 10% aq. NaOH, reflux, o.n. (72%); (v) K_2_CO_3_, Me_2_CO, r.t., o.n. (72–99%); (vi) Pyr, EtOH, reflux, o.n. (53–71%); (vii) *m*-CPBA, DCM, r.t., o.n. (34%); (viii) Nb(OEt)_5_, 30% aq. H_2_O_2_, MeOH, 45 °C, 2 h (87%).

**Scheme 2:**
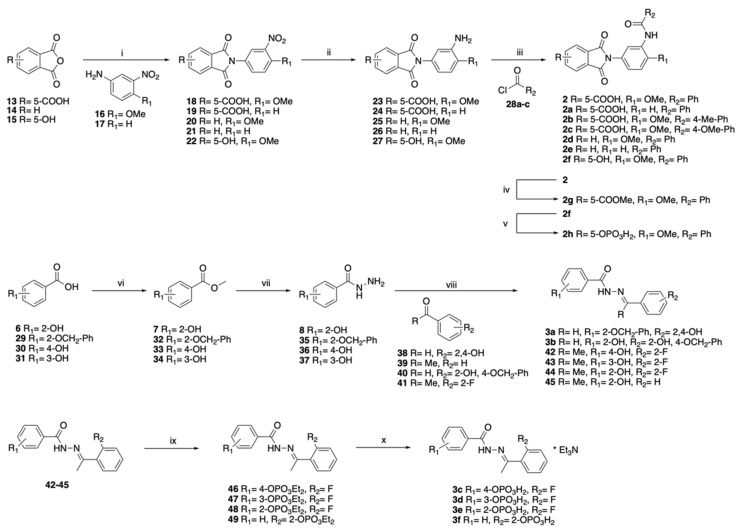
Preparation of **2** and novel target analogues **2a**–**h** and **3a**–**f**. *Reagents and conditions*: (i) AcOH, 130 °C, o.n. (83–93%); (ii) H_2_, Pd/C, THF, r.t., o.n. (74–99%); (iii) Pyr, THF, r.t., o.n. *or* anhydrous DCM, Et_3_N, r.t., o.n. (36–59%); (iv) H_2_SO_4_, MeOH, reflux, o.n. (89%); (v) a. diethyl chlorophosphate, Et_3_N, anhydrous DCM, 0 °C, 2h (65%), b. TMSBr, Pyr, anhydrous DCM, 0 °C to r.t., 56h (32%); (vi) H_2_SO_4_, MeOH, reflux, o.n. *or* MeI, K_2_CO_3_, Me_2_CO, reflux, 4h (84–99%); (vii) NH_2_NH_2_*H_2_O, EtOH, reflux, o.n. (58–96%); (viii) EtOH, reflux, o.n. (47–88%); (ix) diethylchlorophoshpate, Et_3_N, anhydrous DCM, 0 °C to r.t., o.n. (67–99%); (x) TMSBr, Pyr, anhydrous DCM, 0 °C to r.t., o.n. (54–95%).

**Scheme 3:**
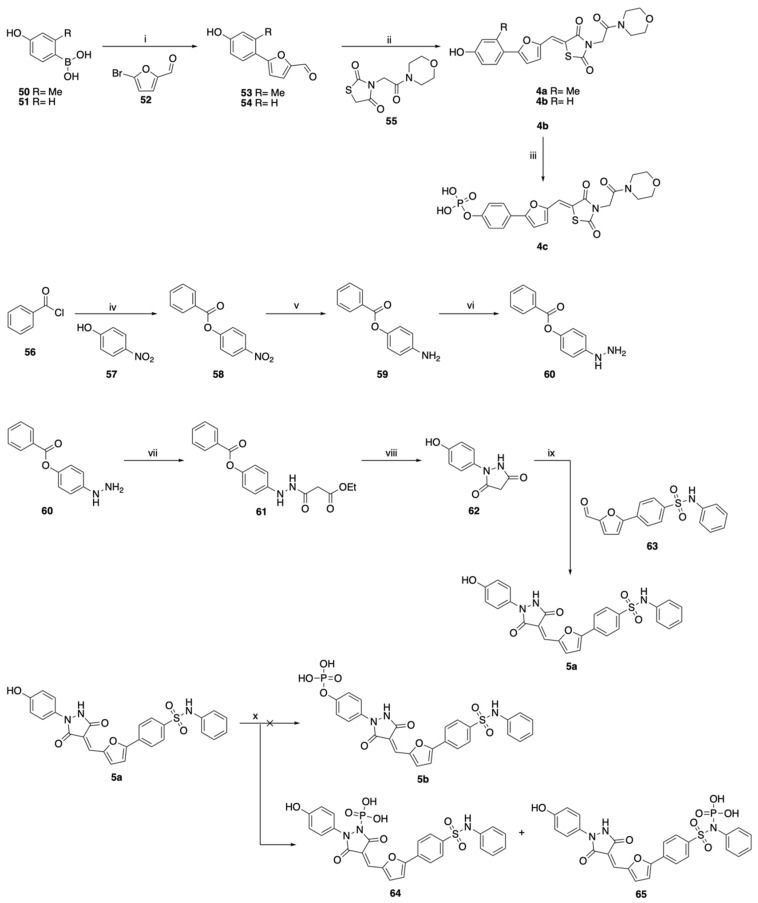
Preparation of novel analogues **4a**–**c** and **5a**. *Reagents and conditions*: (i) Pd(PPh_3_)_4_, K_2_CO_3_, H_2_O, EtOH, PhMe, reflux, o.n. (32–63%); (ii) ß-alanine, AcOH, 100 °C, o.n. (29–67%); (iii) a. diethyl chlorophosphate, Et_3_N, anhydrous DCM, 0 °C, 5h (89%), b. TMSBr, anhydrous DCM, 0 °C to r.t., 24 h (49%); (iv) Pyr, anhydrous DCM, 0 °C to r.t., 20 h (quant.); (v) H_2_, Pd/C, THF, r.t., 24 h (97%); (vi) HCl, NaNO_2_, SnCl_2_, H_2_O, 0 °C, 3 h (77%); (vii) ethyl malonyl chloride, Et_3_N, THF, -10 °C to r.t., 3 h (56%); (viii) 1M NaOH/EtOH, EtOH, r.t., 30 min (73%); (ix) AcOH, 120 °C, 3 h (72%); (x) a. diethyl chlorophosphate, Et_3_N, anhydrous DCM, 0 °C, o.n. (66%), b. TMSBr, anhydrous DCM, 0 °C to r.t., 3 h (58%).

The synthetic preparation of the new target compounds is described in Scheme 1, Scheme 2 and Scheme 3 below.

Scheme 1 shows the synthetic strategy applied for **1** and its analogues **1a**–**f**. 2-Hydroxybenzoic acid **6** was first converted into the corresponding methyl ester **7**, which was treated with hydrazine monohydrate in refluxing EtOH, to afford the corresponding hydrazide **8**, which was then converted into the carbothioamide intermediate **9** in the presence of ammonium isocyanate. The cyclisation of **9** in the presence of 10% aqueous NaOH allowed the formation of 1,2,4-triazole-3-thione intermediate **10**. The reaction between **10** and differently substituted 2-chloroarylacetamides **12a**–**e**, which were obtained by reacting the appropriate aniline **11a**–**e** with 2-chloroacetyl chloride **12** in the presence of potassium carbonate, afforded the desired sulfide compounds **1** and **1a**–**d**. Sulfoxide **1e** and sulfone **1f** were obtained by direct oxidation of **1** in different conditions. An initial attempt to obtain sulfone **1f** was made by reacting **1** with *m*-CPBA in DCM. Although up to 4 equivalents of *m*-CPBA were added and the reaction time was increased up to 5 days, only trace amounts of **1f** were obtained, whilst the main species formed was sulfoxide **1e**, which was isolated and evaluated in the in vitro assays described below. Sulfone **1f** was obtained using 30% aqueous H_2_O_2_ as an oxidizing agent in MeOH, in the presence of niobium pentaethoxide. Following this procedure, the full oxidation of the sulfur atom was completed in two hours, and **1f** was isolated in high yield after chromatographic purification.

Scheme 2 shows the preparation of compounds **2**, **2a**–**h**, and **3a**–**f**.

Compounds **2** and **2a**–**f** were obtained according to a three-step route, starting from anhydrides **13**–**15**, which were converted into the corresponding dioxoisoindolines **18**–**22** through an aminolysis reaction using the differently substituted 3-nitroanilines **16** and **17** in acetic acid. While **13** and **14** were commercially available, **15** was synthesized by dehydration of 4-hydroxyphtalic acid, which was achieved by heating it at 200 °C in the absence of solvent overnight, producing **15** in quantitative yield. The nitro-intermediates **18**–**22** were treated under catalytic hydrogenation conditions, to produce the corresponding amines **23**–**27**, which were reacted with different benzoyl chlorides to afford the desired amides **2** and **2a**–**f**. Lower yields were obtained for those products with a carboxylic acid or hydroxy group in the dioxoisoindoline ring, due to the formation of undesired by-products derived from the reaction between the benzoyl chloride and either the carboxylic acid or the hydroxy group, yielding an anhydride or an ester, respectively. The side reaction was particularly significant for **2c** and **2f**, which could not be obtained using pyridine as a base. Replacement of the base with Et_3_N and the use of anhydrous DCM as a solvent enabled the isolation of **2c** and **2f** in moderate yields. Compound **2g**, the methyl ester of **2**, was obtained via direct esterification of **2**, using H_2_SO_4_ in refluxing MeOH. The hydroxy analogue **2f** was then converted into its corresponding phosphate derivative **2h** by coupling with diethylchlorophosphate, and subsequent deprotection using bromotrimethylsilane in the presence of pyridine.

Compounds **3a**,**b** were obtained according to a three-step route, starting from the differently substituted benzoic acids **6** and **29**, which were first converted into the corresponding methyl esters **7** and **32**, and then treated with hydrazine monohydrate in refluxing EtOH to obtain hydrazides **8** and **35**. These were reacted with differently substituted benzaldehydes **38** and **40**, to afford the target hydrazone products **3a**,**b**. Four additional hydrazones, **42**–**45**, were prepared to obtain suitable substrates for mono-phosphorylation. Intermediates **42**–**45** were coupled with diethylchlorophosphate first, to give diethyl analogues **46**–**49**, which were finally treated with bromotrimethylsilane to afford **3c**–**f**, obtained as triethylamine salts in moderate to good yields after column chromatography purification.

The synthesis of the target new analogues **4a**–**c** and **5a** is described in Scheme 3.

Preparation of **4a**–**c** started with a Suzuki coupling between substituted phenylboronic acids **50**, **51** and 5-bromo-furaldehyde **52** [25], performed using Tetrakis(triphenylphosphine)palladium(0) as catalyst and K_2_CO_3_ as a base, in a mixture of water, ethanol and toluene. While **54** was obtained in good yield after flash column chromatography purification, the yield for **53** was significantly lower, likely due to the presence of the methyl group in *ortho* to the boronic acid functionality, which causes steric hindrance that disadvantages the formation of the new C-C bond. Aldehydes **53** and **54** were then reacted with intermediate **55**, whose preparation has been previously described [18,19], according to a Knoevenagel condensation [26], performed in glacial acetic acid in the presence of β-alanine to afford hydroxy analogues **4a** and **4b**. Compound **4b** was converted into its corresponding phosphate analogue **4c** in two steps, as it was first treated with diethyl chlorophosphate in the presence of triethylamine, and then deprotected using bromotrimethylsilane in anhydrous DCM. Finally, the target hydroxy analogue **5a** was obtained following a six-step route, starting from benzoyl chloride **56**, which was reacted with 4-nitrophenol **57** in anhydrous DCM, in the presence of pyridine as a base, to afford the protected benzoate **58** in quantitative yield. The nitro compound **58** was converted into its corresponding aniline derivative **59** through a catalytic hydrogenation reaction using hydrogen and Pd/C as a catalyst, followed by conversion of **59** into the corresponding diazonium salt, which was reduced in situ using a solution of SnCl_2_ in concentrated HCl, to afford the corresponding hydrazine analogue **60**. Hydrazine **60** was treated with ethyl malonyl chloride to produce intermediate **61**, which underwent cyclisation and removal of the benzoyl protecting group by treatment with sodium hydroxide in EtOH, to provide the cyclic intermediate **62**. In the final reaction step, **62** was reacted with substituted aldehyde **63**, prepared as previously described [18,19], according to another Knoevenagel condensation [26], performed in glacial acetic acid, affording **5a** in good yield after column chromatography purification. Different attempts were made to convert **5a** into its phosphate analogue **5b**, by inserting a diethylphosphate group first, and then deprotecting it using bromotrimethylsilane. After optimizing the reaction conditions to achieve the mono-phosphorylation of substrate **5a**, a preferential phosphorylation of the more reactive nitrogen functionalities at the level of the pyrazolidine-dione (**64**) and sulfonamide (**65**) groups was observed, as confirmed by ^31^P-NMR studies. Phosphorylated by-products **64** and **65** could only be obtained in a ~ 2:1 mixture, which could not be further separated.

### 3.3. Norovirus RdRp Enzymatic Inhibition, Antiviral Activity and Cytotoxicity of the Synthesized Compounds

**Figure 10:**
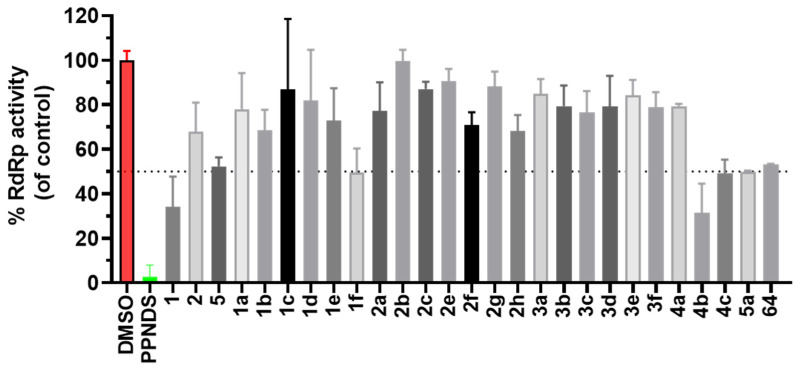
Inhibitory effect of the test compounds on the human norovirus RdRp activity. Compounds were tested at 20 μM. Percentage of inhibition was normalized to control DMSO (red bar). PPNDS (green bar) was used as positive control, along with parent compounds **1, 2** and **5**. The mean values of triplicate datasets with standard error of the mean are shown.

The novel analogues prepared, along with parent compounds **1**, **2** and **5**, were evaluated for their ability to interfere with the activity of recombinant Norovirus RdRp in an in vitro quantitative fluorescence assay [18,19]. All compounds were initially tested at 20 μM in triplicate, compared with the relative activity of mock-treated samples containing the vehicle only (0.5% DMSO [vol/vol]). PPNDS and **5** were used as positive controls. The inhibition observed for the novel compounds at this concentration is summarized in Figure 10.

Most of the new analogues showed at least partial inhibition of the RdRp activity at 20 μM. The best novel compounds found (i.e., **1f**, **4b**, **4c**, **5a** and **64**) exhibited ≥ 50% inhibition of HuNoV RdRp activity at 20 μM (Figure 10). Considering the structural modifications explored for **1**, oxidation of the sulfur linker to sulfoxide (**1e**) is associated with loss of inhibitory activity, in line with the molecular docking results, while sulfone **1f** showed activity retention, which also appears to confirm the in silico predictions. Removal of either one of the two methyl groups (**1a**,**b**) is associated with loss of activity, and the same effect is observed in **1c**,**d**, possibly indicating that the substitution pattern on the methylated phenyl ring of the scaffold is key for inhibition of the RdRp enzyme. Compared to **2**, which only showed limited RdRp inhibition in this assay, the analogues designed to explore structure–activity relationships for this scaffold, **2a**–**c** and **2e**, are all associated with loss of inhibitory activity. Compound **2d** could not be tested due to solubility issues at the test concentration. Analogue **2f** retained inhibitory activity as observed for **2**, suggesting that an electrostatic interaction at the level of this functionality is not key for enzyme inhibition. While analogue **2g** is associated with loss of activity, **2h** retained inhibitory activity and showed the strongest inhibition among the analogues developed for hit **2**. Considering the scaffold of **3**, analogues **3a**,**b** only showed modest inhibition of the RdRp at the test concentration, and similar observations can be extended to the novel phosphate analogues **3c**–**3f**. Taken together, these results indicate that the scaffold of **3** is the least interesting for inhibition of HuNoV of the five hits considered in this study. In our previous study [18], we extensively explored the scaffold of **4**, but none of the developed structural analogues showed any inhibitory activity in the fluorescent assay. Among the compounds developed in this study, **4a** exhibited only a modest inhibition of the RdRp activity at 20 μM, while **4b** showed the best activity in this preliminary assay, indicating that the insertion of a hydroxy substituent in the terminal phenyl ring may be a successful strategy for enhancing the inhibitory profile. Functionalization of this hydroxy group to phosphate in **4c** resulted in a significant RdRp inhibition, although the activity improvement envisaged was not achieved. Finally, considering the scaffold of **5**, which was the best biochemical hit previously identified [18], both analogues **5a** and **64** showed around 50% inhibition of the HuNoV RdRp activity, similar to that obtained for **5**.

For each hit excluding **3**, the analogues that resulted in the highest inhibition percentage of the HuNoV RdRp activity when tested at 20 μM in the fluorescent assay (i.e., **1f, 2a, 4b** and **5a**), were further evaluated in dose–response curves using the same assay. IC_50_ values were then calculated for each test compound and related values are summarized in Table 1. The dose–response curves for the best analogues are reported in the Supplementary Information (Figure S1).

**Figure 11:**
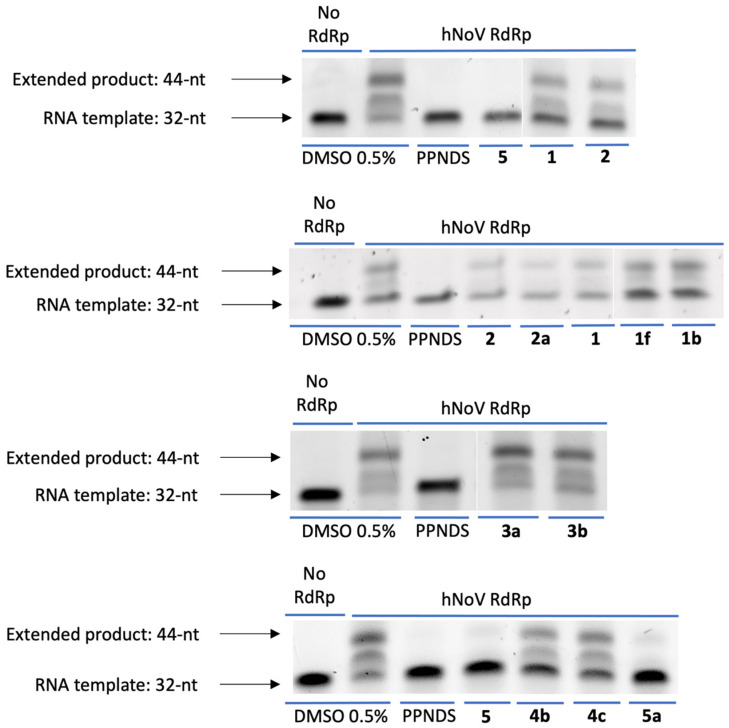
Counter-screen gel-shift assay to confirm HuNoV (GII.4) RdRp inhibitory activity for compounds **1**, **1b**, **1f**, **2**, **2a**, **3a**,**b**, **4b**,**c**, **5** and **5a**. The compounds were examined for inhibition of primed elongation activity. PE44-NoV RNA templates (32 nucleotides) were extended (44 nucleotides) by the RdRp in the absence of any test compounds (0.5% DMSO [vol/vol] negative control) or with test compounds at a fixed concentration of 100 μM. PPNDS was used as positive controls (100 μM) to demonstrate complete inhibition, and no RdRp was used as a negative control.

Considering the data obtained by the fluorescent inhibition assay, **4b** and **5a** emerged as the best polymerase inhibitors found among the new structural analogues synthesized. Interestingly, both these compounds are hydroxy-analogues of two of the original hits (**4** and **5**, respectively), while the new compounds carrying an additional phosphate group (**2**, **3c**–**f**, **4c**), with the exception of **64**, did not show the expected improvement in inhibition of the viral RdRp. To confirm the RdRp inhibitory activity observed in the fluorescence RdRp assay and exclude false positives, compounds **1**, **1b**, **1f**, **2**, **2a**, **3a**,**b**, **4b**,**c**, **5** and **5a** were also tested in a gel-shift RdRp assay, at a fixed concentration of 100 μM, following a previously reported procedure [18,19]. PPNDS was used as positive control, as it showed a complete inhibition of RdRp-based transcription at 100 μM (no extension of 32 nucleotide RNA template (PE44-NoV) to 44 nucleotides in the presence of an active RdRp). The results obtained for this assay are summarized in Figure 11, while images of the full gels are reported in the Supporting Information (Figure S2). 

From the gel-shift assay (Figure 11), hit **5** confirmed to completely abolish HuNoV RdRp activity at 100 μM, while the other two hits tested, **1** and **2**, caused only partial inhibition of the RdRp activity. While this result is in line with the activity observed in the fluorescence inhibition assay (Table 1) for compound **1**, an increased effect was expected for compound **2** at 100 μM, as this compound exhibited an IC_50_ 41.9 μM in the fluorescent assay. Novel analogues **3a** and **3b** did not show significant inhibition of the RdRp activity in the gel-shift assay, while **1b**, **1f** and **4c** showed only a partial inhibition. While a significant but partial inhibition of the formation of the extended RNA product was observed for **4b**, a nearly complete inhibition was observed for **5a**, which showed an analogous profile to its parent compound **5**. Considering the results of both assays combined, novel compounds **4b** and **5a** are confirmed as the best HuNoV RdRp inhibitors found within this series of newly developed analogues.

To explore their potential interference with norovirus replication in cell-based systems, compounds **1**, **1a**–**d**, **1f**, **2**, **2a**–**f**, **3**, **3a**,**b**, **4**, **4b**, **5** and **5a** were evaluated against the genogroup V mouse virus (MNV) in the mouse macrophage cell line RAW264.7. The novel phosphate analogues **2h**, **3c**–**f**, **4c** and **64** were excluded from this assay, as these compounds would require masking of the phosphate group with a pro-drug functionality to enable cell permeation and cell-based evaluations. In this assay, compounds are screened for their ability to protect infected cells from the virus-induced cytopathic effect. Compounds identified using this experimental setting have shown activity against HuNoV in vitro and in vivo, as for example nucleoside RdRp inhibitor 2′-C-methylcytidine (2CMC) [24,27], which was used as positive control. The test compounds were evaluated at eight different concentrations in the 0.6–100 μM range, both for their ability to reduce the virus-induced cytopathic effect, and for potential cytotoxicity. Although the new compounds tested did not show significant inhibition of the MNV-induced cytopathic effect at the test concentrations (Supporting Information, Table S1), relevant results were obtained for their toxicity in the cell line used. As shown in Table S1, all the analogues tested with the scaffold of previous hit **1** were associated with a cytotoxic effect at the test concentrations, with CC_50_ values in the micromolar range. The only exception to this trend is represented by **1f**, which is not associated with any cytotoxicity at concentrations up to 100 μM. Combining this improvement with the observed inhibition of the HuNoV RdRp in both biochemical assays, **1f** can be regarded as a suitable starting point for further structural optimization. The scaffolds of **3** and **5** were also associated with a cytotoxic effect observed at the test concentrations, more prominent for compound **5a**, which displayed a CC_50_ value in the low micromolar range. While original hit **4** did not display any cytotoxicity, **4b** showed a cytotoxic effect in the micromolar range. Interestingly, the scaffold of **2** did not appear to be associated with cytotoxicity. Although a significant inhibition of the RdRp activity could not be confirmed in both the fluorescence and the gel-shift assay for any of the compounds based on the structure of **2**, the RdRp inhibition data obtained for **4b** and **5a** appears to indicate the suitability of these compounds as starting points for further structural optimization efforts, to improve the RdRp inhibition and most importantly to achieve an antiviral effect against norovirus replication in cell-based assays.

## 4. Conclusions

The identification of antiviral agents to be used as treatments and prophylactic measures for norovirus outbreaks is urgently needed. Due to its essential role for the virus replication and its conservation among different genotypes, the viral polymerase is a promising target for the identification of direct-acting antivirals. In this study, we have designed and synthesized different series of novel analogues, starting from the structures of five biochemical hits, previously identified as inhibitors of the human norovirus polymerase. Following optimization of multiple-step synthetic routes for the diverse structural families explored, 25 new compounds were prepared and evaluated in biochemical assays, to assess direct inhibition of the viral enzyme, and cell-based studies, to investigate antiviral properties and cytotoxicity. While most of the new compounds showed at least a partial inhibition of the viral RdRp at micromolar concentrations, different new analogues displayed retained or improved inhibition of the enzyme compared with the parent hit compounds. Although none of the novel analogues showed a significant inhibition of the replication of murine norovirus in cells, different chemotypes were confirmed as inhibitors of the RdRp in vitro, representing useful starting points for further optimization efforts.

## Figures and Tables

**Table 1:** IC_50_ values and structures of the novel compounds against HuNoV (GII.4) RdRp activities.

Compound	Structure	In Vitro RdRp Activity Assay IC_50_ (μM) ^1^
**1f**	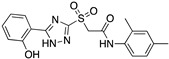	>100
**2**	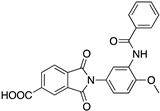	41.9 ± 0.6
**2a**	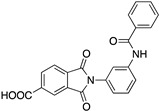	>100
**4**	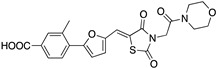	15.0 ^2^
**4b**	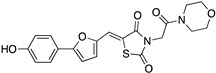	15.6 ± 1.8
**5**	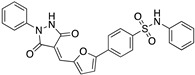	5.6 ^2^
**5a**	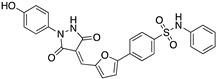	17.3 ± 1.2
**PPNDS**	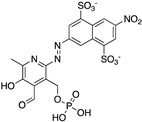	1.5 ± 0.25 ^2^

^1^ IC_50_ values correspond to the compound concentrations that inhibited the HuNoV activity in vitro by 50%. Data shown are the mean ± standard deviations of at least 2 independent experiments conducted in triplicate. ^2^ Activity data as previously reported [18,19].

## Data Availability

Not applicable.

## Supplementary Materials

The following supporting information can be downloaded at: https://www.mdpi.com/article/10.3390/v15010074/s1, Preparation and characterization of synthetic intermediates; Preparation and characterization of final products; Figure S1: Dose–response curves for compounds **4b** and **5a**; Figure S2: Full gels for the gel-shift confirmatory assay; Table S1: Antiviral and cytotoxicity data for selected compounds; Figure S3: 2D interaction diagrams for the docking results.
