# Supplementary material for: Structural Investigations on Novel Non-Nucleoside Inhibitors of Human Norovirus Polymerase
Source: Viruses. 2022 Dec 27;15(1):74. doi: 10.3390/v15010074 (PMC9864251; doi:10.3390/v15010074)
Supplement: Supplementary file 1 [file viruses-15-00074-s001.zip › viruses-2089883-supplementary.pdf]

Supplementary Information

Contents

Page S2 Preparation and characterisation of synthetic intermediates

Page S10 Preparation and characterisation of final products

Page S21 Figure S1. Dose-response curves for compounds **4b** and **5a**

Page S22 Figure S2. Full gels for the gel-shift confirmatory assay

Page S24 Table S1. Antiviral and cytotoxicity data for selected compounds

Page S26 Figure S3. 2D interaction diagrams for the docking results.

Page S25 References

Preparation and characterisation of synthetic intermediates

1. Synthesis of methyl 2-hydroxybenzoate 7

A mixture of 2-hydroxybenzoic acid **6** (7.24 mmol, 1 eq.), sulfuric acid (7 mL) and methanol (30 mL) was heated at reflux overnight. The solvent was evaporated under *vacuum*. Water (20 mL) was added and the mixture was neutralised to pH 6-7 using solid sodium bicarbonate. The water layer was extracted with DCM (3x 20 mL). The combined organic layers were collected, dried over $MgSO_4$ and evaporated under *vacuum*. The crude products were purified by automated flash column chromatography eluting with *n*-hexane:EtOAc 90:10 v/v increasing to *n*-hexane:EtOAc 0:100 v/v in 10 CV. Obtained as a colourless oil in 88% yield. ^{1}H-NMR ($CDCl_3$), δ: 3.98 (s, 3H), 6.89-6.92 (m, 1H), 7.01 (dd, J_1= 8.4 Hz, J_2= 1.1 Hz, 1H), 7.46-7.50 (m, 1H), 7.86 (dd, J_1= 8.0 Hz, J_2= 1.6 Hz, 1H), 10.77 (bs, 1H). ^{13}C-NMR ($CDCl_3$), δ: 52.2, 112.4, 117.4, 119.3, 130.3, 135.7, 162.0, 170.3.

Methyl esters **33-34** were synthesised according to the same procedure.

2. General procedure for the synthesis of 2-hydroxybenzohydrazides 8, 35-37

The appropriate ester **7**, **32-34** (5.0 mmol, 1 eq.) was diluted in 12 mL of EtOH and hydrazine monohydrate (15.0 mmol, 3 eq.) was added to the mixture. The reaction was refluxed overnight, then cooled to room temperature and the precipitate formed was collected by filtration, washed with thoroughly cold EtOH and *n*-hexane, and dried under *vacuum*.

• 2-Hydroxybenzohydrazide 8

A precipitate was not obtained for this reaction, therefore the solvent was evaporated under *vacuum*, and the residue was partitioned between EtOAc (3x 20 mL) and water (15 mL). The combined organic layers were dried over $MgSO_4$ and concentrated at reduced pressure to give the title compound as an off-white solid in 94% yield. ^{1}H-NMR ($CDCl_3$), δ: 4.67 (bs, 2H), 6.84-6.87 (m, 1H), 6.90 (dd, J_1= 8.3 Hz, J_2= 0.8 Hz, 1H), 7.36-7.39 (m, 1H), 7.80 (dd, J_1= 8.2 Hz, J_2= 1.6 Hz, 1H), 10.04 (bs, 1H), 12.47 (bs, 1H). ^{13}C-NMR ($CDCl_3$), δ: 114.8, 117.7, 119.1, 127.5, 133.8, 160.0, 168.3.

Benzohydrazides **36-37** were synthesised according to the same procedure.

• 2-(Benzyloxy)benzohydrazide 35

Obtained as a white solid in 58% yield. ^{1}H-NMR ($CDCl_3$), δ: 4.14 (bs, 2H), 5.24 (s, 2H), 7.04-7.06 (m, 1H), 7.11-7.14 (m, 1H), 7.38-7.42 (m 1H), 7.43-7.45 (m, 3H), 7.46-7.48 (m, 2H), 8.25 (dd, J_1= 8.0 Hz, J_2= 2.0 Hz, 1H).

3. Synthesis of 2-(2-hydroxybenzoyl)hydrazine-1-carbothioamide 9

2-Hydroxybenzohydrazide **8** (4.40 mmol, 1 eq.) was dissolved in EtOH (23 mL) and the mixture was stirred at room temperature for 5 minutes. Ammonium thiocyanate (4.84 mmol, 1.1 eq.) was added, followed by concentrated HCl (1 mL). The solution was heated to reflux for 24 hours. The reaction was cooled to room temperature, the precipitate formed was filtered off and the filtrate was dried at reduced

pressure. The crude product was purified by recrystallisation from EtOH to give the title compound as a white solid in 58% yield. ^{1}H-NMR (DMSO-d$_6$), δ: 6.89-6.95 (m, 2H), 7.41-7.45 (m, 1H), 7.68-7.83 (m, 2H), 7.89-7.95 (m, 1H), 9.46 (bs, 1H), 10.54 (bs, 1H), 11.88 (bs, 1H). ^{13}C-NMR (DMSO-d$_6$), δ: 115.4, 117.6, 119.2, 128.9, 134.5, 159.9, 169.1, 176.7.

4. Synthesis of 5-(2-hydroxyphenyl)-2,4-dihydro-3*H*-1,2,4-triazole-3-thione 10

A mixture of 2-(2-hydroxybenzoyl)hydrazine-1-carbothioamide **9** (1.17 mmol, 1 eq.) and a 10% aqueous NaOH solution (15 mL) was heated under reflux for 7 hours. The reaction was then cooled to room temperature and acidified using a 10% aqueous HCl solution (8 mL). The precipitate formed was collected by filtration, washed thoroughly with deionised water, and dried under *vacuum* to give the title compound as a white solid in 72% yield. ^{1}H-NMR (DMSO-d$_6$), δ: 6.91-6.95 (m, 1H), 7.00 (dd, J_1= 8.2 Hz, J_2= 0.8 Hz, 1H), 7.33-7.37 (m, 1H), 7.64 (dd, J_1= 7.7 Hz, J_2= 1.6 Hz, 1H), 10.22 (bs, 1H), 13.22 (bs, 1H), 13.69 (bs, 1H). ^{13}C-NMR (DMSO-d$_6$), δ: 112.4, 117.0, 120.0, 128.8, 132.5, 149.7, 156.3, 166.6.

5. General procedure for the synthesis of 2-chloro-*N*-arylacetamides 12a-e

2-Chloroacetyl chloride (1.2 mmol, 1.2 eq.) was added dropwise to a mixture of the differently substituted anilines **11a-e** (1 mmol, 1 eq.) and K_2CO_3 (2mmol, 2 eq.) in acetone (4 mL). The reaction was stirred at room temperature overnight. The mixture was then quenched with H_2O (10 mL) and extracted with EtOAc (3x 20 mL). The combined organic layers were washed with H_2O (10 mL) and brine (10 mL), dried over Na_2SO_4 and concentrated under *vacuum*. The crude residues were purified by recrystallisation from EtOH.

- **2-Chloro-*N*-(*o*-tolyl)acetamide 12a**

Obtained as a white solid in 80% yield. ^{1}H-NMR (DMSO-d$_6$), δ: 2.21 (s, 3H), 4.30 (s, 2H), 7.10-7.14 (m, 1H), 7.16-7.20 (m, 1H), 7.22-7.24 (m, 1H), 7.37-7.40 (m, 1H), 9.64 (bs, 1H). ^{13}C-NMR (DMSO-d$_6$), δ: 18.3, 43.5, 125.5, 126.1, 126.8, 130.8, 132.5, 136.1, 165.34.

- **2-Chloro-*N*-(*p*-tolyl)acetamide 12b**

Obtained as a white solid in 99% yield. ^{1}H-NMR (DMSO-d$_6$), δ: 2.26 (s, 3H), 4.22 (s, 2H), 7.14 (d, J= 8.1 Hz, 2H), 7.46 (d, J= 8.1 Hz, 2H), 10.19 (bs, 1H). ^{13}C-NMR (DMSO-d$_6$), δ: 20.9, 44.0, 119.9, 129.7, 133.4, 136.6, 165.0.

- **2-Chloro-*N*-(4-(trifluoromethyl)phenyl)acetamide 12c**

Obtained as a white solid in 98% yield. ^{1}H-NMR (DMSO-d$_6$), δ: 4.31 (s, 2H), 7.70 (d, J= 8.5 Hz, 2H), 7.79 (d, J= 8.1 Hz, 2H), 10.65 (bs, 1H). ^{19}F-NMR (DMSO-d$_6$), δ: -60.43. ^{13}C-NMR (DMSO-d$_6$), δ: 44.0, 119.7, 124.1, 125.8, 126.6, 142.5, 165.7.

- **2-Chloro-*N*-(3-chloro-4-methylphenyl)acetamide 12d**

Obtained as a white solid in 72% yield. ^{1}H-NMR (DMSO-d_6), δ: 2.28 (s, 3H), 4.24 (s, 2H), 7.30 (d, J= 8.6 Hz, 1H), 7.36 (dd, J_1= 8.6 Hz, J_2= 2.0 Hz, 1H), 7.77 (d, J= 2.0 Hz, 1H), 10.38 (bs, 1H). ^{13}C-NMR (DMSO-d_6), δ: 19.5, 43.9, 118.4, 119.7, 131.0, 131.7, 133.5, 138.0, 165.2.

• **2-Chloro-*N*-(2,4-dimethylphenyl)acetamide 12e**

Obtained as a white solid in 99% yield. ^{1}H-NMR ($CDCl_3$), δ: 2.18 (s, 3H), 2.22 (s, 3H), 4.15 (s, 2H), 6.94-6.97 (m, 2H), 7.60 (d, J= 7.9 Hz, 1H), 8.06 (bs, 1H). ^{13}C-NMR ($CDCl_3$), δ: 17.0, 20.9, 122.8, 127.4, 129.3, 131.2, 132.0, 135.6, 163.9.

6. Synthesis of 5-hydroxyisobenzofuran-1,3-dione 15

4-Hydroxyphtalic acid (5.5 mmol, 1 eq.) was heated at 200 °C overnight in the absence of solvent. The resulting material was cooled to room temperature and the solid formed was washed thotoughly with EtOAc to obtain the title compound as an off-white solid in quantitative yield. ^{1}H-NMR (DMSO-d_6), δ: 7.27 (dd, J_1= 2.2 Hz, J_2= 0.5 Hz, 1H), 7.29 (dd, J_1= 8.3 Hz, J_2= 2.2 Hz, 1H), 7.91 (dd, J_1= 8.3 Hz, J_2= 0.5 Hz, 1H), 11.43 (bs, 1H).

7. General procedure for the synthesis of (3-nitrophenyl)-1,3-dioxoisoindolines 18-22

A mixture of the appropriate isobenzofuran-1,3-dione **13-15** (5.2 mmol, 1 eq.) and the differently substituted 3-nitroaniline **16-17** (5.2 mmol, 1 eq.) in glacial acetic acid (20 mL) was stirred at 130 °C overnight. The reaction was cooled to room temperature, and the resulting precipitate was isolated by *vacuum* filtration, washed with deionised water (3x 15 mL) and dried in *vacuo*.

• **2-(4-Methoxy-3-nitrophenyl)-1,3-dioxoisoindoline-5-carboxylic acid 18**

Obtained as a yellow solid in 93% yield. ^{1}H-NMR (DMSO-d_6), δ: 4.01 (s, 3H), 7.56 (d, J= 9.0 Hz, 1H), 7.80 (dd, J_1= 9.0 Hz, J_2= 2.5 Hz, 1H), 8.07 (d, J= 2.5 Hz, 1H), 8.10 (d, J= 8.0 Hz, 1H), 8.30 (s, 1H), 8.43 (dd, J_1= 8.0 Hz, J_2= 1.3 Hz, 1H), 13.70 (bs, 1H). ^{13}C-NMR (DMSO-d_6), δ: 57.6, 115.4, 123.9, 124.3, 124.3, 124.4, 132.5, 134.0, 135.3, 136.0, 137.0, 139.1, 152.2, 162.2, 166.5, 166.5.

• **2-(3-Nitrophenyl)-1,3-dioxoisoindoline-5-carboxylic acid 19**

Obtained as a white solid in 87% yield. ^{1}H-NMR (DMSO-d_6), δ: 7.85-7.89 (m, 1H), 7.97-7.99 (m, 1H), 8.13 (dd, J_1= 7.7 Hz, J_2= 0.6 Hz, 1H), 8.31-8.34 (m, 1H), 8.35-8.37 (m, 1H), 8.42-8.43 (m, 1H), 8.44 (dd, J_1= 7.8 Hz, J_2= 1.3 Hz, 1H), 13.83 (bs, 1H). ^{13}C-NMR (DMSO-d_6), δ: 122.4, 123.3, 124.0, 124.4, 130.9, 132.5, 133.3, 135.3, 134.1, 136.1, 137.1, 166.2, 166.3, 166.3.

• **2-(4-Methoxy-3-nitrophenyl)isoindoline-1,3-dione 20**

Obtained as a yellow solid in 92% yield. ^{1}H-NMR (DMSO-d_6), δ: 4.00 (s, 3H), 7.54 (d, J= 9.0 Hz, 1H), 7.79 (dd, J_1= 9.0 Hz, J_2= 2.5 Hz, 1H), 7.92-7.93 (m, 2H), 7.98-7.99 (m, 2H), 8.05 (d, J= 2.5 Hz, 1H). ^{13}C-NMR (DMSO-d_6), δ: 57.5, 115.3, 123.9, 124.3, 124.4, 124.5, 132.0, 134.1, 135.2, 152.1, 167.3, 178.7.

• **2-(3-Nitrophenyl)isoindoline-1,3-dione 21**

Obtained as an off-white solid in 83% yield. ^{1}H-NMR (DMSO-d_6), δ: 7.84-7.87 (m, 1H), 7.93-7.94 (m, 1H), 7.95-7.96 (m, 1H), 7.97-7.99 (m, 1H), 8.01-8.02 (m, 1H), 8.03-8.04 (m, 1H), 8.29-8.32 (m, 1H), 8.41-8.43 (m, 1H). ^{13}C-NMR (DMSO-d_6), δ: 122.4, 123.2, 124.0, 130.7, 132.0, 133.5, 134.1, 135.3, 148.2, 167.0.

- **5-Hydroxy-2-(4-methoxy-3-nitrophenyl)isoindoline-1,3-dione 22**

Obtained as a yellow solid in 84% yield. ^{1}H-NMR (DMSO-d_6), δ: 3.99 (s, 3H), 7.20 (dd, J_1= 8.2 Hz, J_2= 2.2 Hz, 1H), 7.23 (dd, J_1= 1.8 Hz, J_2= 0.3 Hz, 1H), 7.52 (d, 1H, J= 9.0 Hz), 7.75 (dd, J_1= 9.0 Hz, J_2= 2.5 Hz, 1H), 7.80 (dd, J_1= 8.5 Hz, J_2= 0.3 Hz, 1H), 8.02 (d, J= 2.5 Hz, 1H), 11.08 (bs, 1H). ^{13}C-NMR (DMSO-d_6), δ: 57.5, 110.4, 115.2, 121.3, 122.1, 124.3, 124.7, 126.2, 133.9, 134.7, 139.2, 151.9, 167.0, 167.1.

8. General procedure for the synthesis of (3-amino)-1,3-dioxoisoindolines 23-27

10% wet Pd/C (10% w/w) was added to a solution of the appropriate (3-nitro)-1,3-dioxoisoindoline **18-22** (2 mmol, 1 eq.) in anhydrous THF (25 mL). The reaction was stirred at room temperature overnight under a hydrogen atmosphere. The catalyst was then removed by filtration through a celite pad, and the solvent was evaporated under reduced pressure. The crude residue was purified by recrystallisation from DCM.

- **2-(3-Amino-4-methoxyphenyl)-1,3-dioxoisoindoline-5-carboxylic acid 23**

Obtained as a light brown solid in 74% yield. ^{1}H-NMR (DMSO-d_6), δ: 3.84 (s, 3H), 6.51 (d, J= 2.3 Hz, 1H), 6.58 (dd, J_1= 8.0 Hz, J_2= 2.3 Hz, 1H), 6.90 (d, J= 8.4 Hz, 1H), 8.03-8.06 (m, 1H), 8.28-8.30 (m, 1H), 8.41 (dd, J_1= 7.6 Hz, J_2= 1.1 Hz, 1H), 13.75 (bs, 1H).

- **2-(3-Aminophenyl)-1,3-dioxoisoindoline-5-carboxylic acid 24**

Obtained as a yellow solid in 93% yield. ^{1}H-NMR (DMSO-d_6), δ: 6.52-7.55 (m, 1H), 6.59-60 (m, 1H), 6.61-6.64 (m, 1H), 7.12-7.15 (m, 1H), 8.05 (dd, J_1= 7.7 Hz, J_2= 0.6 Hz, 1H), 8.27-8.29 (m, 1H), 8.40 (dd, J_1= 7.7 Hz, J_2= 1.4 Hz, 1H). ^{13}C-NMR (DMSO-d_6), δ: 113.0, 114.1, 114.8, 123.7, 124.1, 129.5, 132.5, 132.7, 135.3, 135.8, 136.8, 149.7, 159.2, 166.3, 166.8.

- **2-(3-Amino-4-methoxyphenyl)isoindoline-1,3-dione 25**

Obtained as a yellow solid in 99% yield. ^{1}H-NMR (DMSO-d_6), δ: 3.87 (s, 3H), 4.99 (s, 2H), 6.60 (dd, J_1= 8.4 Hz, J_2= 2.2 Hz, 1H), 6.69 (d, J= 2.2 Hz, 1H), 6.94 (d, J= 8.4 Hz, 1H), 7.93-7.96 (m, 2H), 7.97-7.99 (m, 2H). ^{13}C-NMR (DMSO-d_6), δ: 56.0, 110.7, 113.0, 115.5, 123.7, 124.1, 132.0, 135.0, 138.4, 146.5, 167.8.

- **2-(3-Aminophenyl)isoindoline-1,3-dione 26**

Obtained as a yellow solid in 98% yield. ^{1}H-NMR (DMSO-d_6), δ: 5.26 (bs, 2H), 6.51-6.54 (m, 1H), 6.58-6.60 (m, 1H), 6.61-6.64 (m, 1H), 7.11-7.14 (m, 1H), 7.88-7.91 (m, 2H), 7.93-7.96 (m, 2H). ^{13}C-NMR (DMSO-d_6), δ: 113.1, 114.0, 115.0, 123.8, 129.5, 132.0, 132.9, 135.0, 149.7, 167.5.

- **2-(3-Amino-4-methoxyphenyl)-5-hydroxyisoindoline-1,3-dione 27**

Obtained as a yellow solid in 77% yield. ^{1}H-NMR (DMSO-d_6), δ: 3.81 (s, 3H), 4.91 (bs, 2H), 6.50 (dd, J_1= 8.5 Hz, J_2= 2.3 Hz, 1H), 6.59 (d, 1H, J= 2.3 Hz, H-aromatic), 6.87 (d, 1H, J= 8.5 Hz, H-aromatic), 7.12-7.17

(m, 2H), 7.74 (d, J= 8.1 Hz, 1H), 11.10 (bs, 1H). ^{13}C-NMR (DMSO-d_6), δ: 56.0, 110.4, 110.8, 113.1, 115.5, 121.2, 121.6, 125.4, 125.9, 134.8, 138.4, 146.4, 164.5, 167.6, 167.7.

9. Synthesis of methyl 2-(benzyloxy)benzoate 32

K_2CO_3 (10.95 mmol, 2.5 eq.) and CH_3I (6.57 mmol, 1.5 eq.) were added to a solution of 2-(benzyloxy)benzoic acid **29** (4.38 mmol, 1 eq.) in acetone (30 mL). The reaction was heated at reflux for 4 hours, then cooled to room temperature. The precipitate formed was filtered off, and the filtrate was concentrated under reduced pressure to give the title compound as a white solid in 99% yield. ^{1}H-NMR ($CDCl_3$), δ: 3.93 (s, 3H), 5.22 (s, 2H), 7.00-7.02 (m, 1H), 7.03-7.05 (m, 1H), 7.31-7.35 (m 1H), 7.39-7.43 (m, 2H), 7.44-7.47 (m, 1H), 7.51-7.53 (m, 2H), 7.85 (dd, J_1= 7.6 Hz, J_2= 1.6 Hz, 1H). ^{13}C-NMR ($CDCl_3$), δ: 52.0, 70.6, 113.9, 120.6, 120.8, 126.8, 127.7, 128.5, 131.7, 133.4, 136.8, 158.1, 166.8.

10. General procedure for the synthesis of benzohydrazones 42-45

The appropriate benzaldehyde of acetophenone **39**, **41** (1.1 mmol, 1.1 eq.) was added to a solution of the differently substituted benzohydrazide **8**, **36-37** (1 mmol, 1 eq.) in EtOH (7 mL). The mixture was stirred at reflux temperature overnight, then it was cooled to room temperature, and the precipitate formed was collected by filtration and washed with cold EtOH. The crude residue was purified by recrystallization from EtOH.

• ***N'*-(1-(2-Fluorophenyl)ethylidene)-4-hydroxybenzohydrazide 42**

Obtained as a white solid in 47% yield. UPLC-MS (Method C): t_R 1.71 min, MS [ESI, m/z]: 273.0 $[M+H]^+$.

• ***N'*-(1-(2-Fluorophenyl)ethylidene)-3-hydroxybenzohydrazide 43**

Obtained as a white solid in 56% yield. UPLC-MS (Method B): t_R 2.51 min, MS [ESI, m/z]: 273.1 $[M+H]^+$.

• ***N'*-(1-(2-Fluorophenyl)ethylidene)-2-hydroxybenzohydrazide 44**

Obtained as a white solid in 62% yield. ^{1}H-NMR (DMSO-d_6), δ: 2.33-2.34 (m, 3H), 6.98-7.05 (m, 2H), 7.28-7.32 (m, 2H), 7.41-7.51 (m, 2H), 7.62-7.65 (m, 1H), 7.98-8.0 (m, 1H), 11.41 (bs, 1H), 11.79 (bs, 1H). UPLC-MS (Method C): t_R 1.87 min, MS [ESI, m/z]: 273.0 $[M+H]^+$.

• ***N'*-(1-(2-Fluorophenyl)ethylidene)-2-hydroxybenzohydrazide 45**

Obtained as a white solid in 75% yield. ^{1}H-NMR (DMSO-d_6), δ: 2.49 (s, 3H), 6.90-7.93 (m, 2H), 7.30-7.33 (m, 1H), 7.56.90-7.93 (m, 2H), 4-7.57 (m, 2H), 7.95 (d, J = 7.3 Hz, 2H), 11.33 (bs, 1H), 13.36 (bs, 1H). UPLC-MS (Method C): t_R 1.99 min, MS [ESI, m/z]: 255.1 $[M+H]^+$.

11. General procedure for the synthesis of diethylphosphates 46-49

The appropriate hydroxybenzohydrazide **42-45** (0.4 mmol, 1 eq.) was suspended in dry DCM (2 mL). After five minutes, Et_3N (0.55 mmol, 1.5 eq.) was added at room temperature. The solution was then cooled to 0 °C and diethylchlorophosphate (0.44 mmol, 1.2 eq.) was added in one portion. The mixture was stirred at room temperature overnight. The solvent was removed under *vacuum* and the crude

product was purified by automated flash column chromatography eluting with *n*-hexane:AcOEt 0:100 v/v increasing to *n*-hexane:AcOEt 50:50 v/v in 15 CV.

• **Diethyl (4-(2-(1-(2-fluorophenyl)ethylidene)hydrazine-1-carbonyl)phenyl) phosphate 46**

Obtained as a colourless oil in 67% yield. ^{1}H-NMR (DMSO-d_6), δ: 1.27-1.30 (m, 6H), 2.50-2.51 (m, 3H), 4.14-4.22 (m, 4H), 7.24-7.34 (m, 4H), 7.43-7.65 (m, 2H), 7.93-7.94 (m, 2H), 10.83 (bs, 1H). UPLC-MS (Method C): t_R 1.94 min, MS [ESI, m/z]: 409.2 $[M+H]^+$.

• **Diethyl (3-(2-(1-(2-fluorophenyl)ethylidene)hydrazine-1-carbonyl)phenyl) phosphate 47**

Obtained as a colourless oil in 99% yield. ^{1}H-NMR (DMSO-d_6), δ: 1.27-1.29 (m, 6H), 2.35-2.37 (m, 3H), 4.15-4.19 (m, 4H), 7.28-7.30 (m, 2H), 7.44-7.76 (m, 6H), 10.91 (bs, 1H). UPLC-MS (Method C): t_R 1.96 min, MS [ESI, m/z]: 409.2 $[M+H]^+$.

• **Diethyl (2-(2-(1-(2-fluorophenyl)ethylidene)hydrazine-1-carbonyl)phenyl) phosphate 48**

Obtained as a colourless oil in 84% yield. ^{1}H-NMR (DMSO-d_6), δ: 1.16-1.92 (m, 6H), 2.27-2.33 (m, 3H), 4.01-4.07 (m, 4H), 7.07-7.19 (m, 1H), 7.25-7.36 (m, 3H), 7.40-7.50 (m, 2H), 7.55-7.66 (m, 2H), 10.88 (bs, 1H). UPLC-MS (Method C): t_R 1.99 min, MS [ESI, m/z]: 409.2 $[M+H]^+$.

• **2-(1-(2-Benzoylhydrazineylidene)ethyl)phenyl diethyl phosphate 49**

Obtained as a colourless oil in 90% yield. ^{1}H-NMR (DMSO-d_6), δ: 1.13-1.18 (m, 6H), 2.22-2.28 (m, 3H), 4.07-4.10 (m, 4H), 7.21-7.37 (m, 4H), 7.40-7.51 (m, 4H), 7.77-7.80 (m, 1H), 10.68 (bs, 1H). UPLC-MS (Method C): t_R 1.89 min, MS [ESI, m/z]: 391.2 $[M+H]^+$.

12. General procedure for the synthesis of furaldehydes 53-54

A solution of K_2CO_3 (5.4 mmol, 1.5 eq.) in deionised water (7.5 mL) was added to a mixture of the appropriate phenylboronic acid **50-51** (3.6 mmol, 1 eq.) and 5-bromo-2-furaldehyde **52** [1] (4.4 mmol, 1.2 eq.) in toluene/ethanol 1:1 v/v (15 mL /15 mL) . Tetrakis(triphenylphosphine)palladium(0) (0.040 g) was added. The reaction mixture was stirred at 90°C overnight, then cooled to room temperature and concentrated under reduced pressure. The residue was extracted between DCM (3x 75 mL) and water (40 mL). The combined organic layers were washed with brine (2x 10 mL), dried over Na_2SO_4 and evaporated under *vacuum*. The crude residue was purified by automated flash column chromatography eluting with *n*-hexane:AcOEt 0:100 v/v increasing to *n*-hexane:AcOEt 0:100 v/v in 10 CV.

• **5-(4-Hydroxy-2-methylphenyl)furan-2-carbaldehyde 53**

Obtained as a brown solid in 32% yield. ^{1}H-NMR (DMSO-d_6), δ: 2.43 (s, 3H), 6.73-6.74 (m, 2H), 6.87 (d, J=3.7 Hz, 1H), 7.59-7.60 (m, 1H), 7.61 (d, J= 3.7 Hz, 1H), 9.55 (bs, 1H), 9.89 (s, 1H). ^{13}C-NMR (DMSO-d_6), δ: 21.7, 110.0, 113.8, 115.4, 118.3, 119.4, 129.7, 137.7, 151.5, 158.8, 159.2, 176.3.

• **5-(4-Hydroxyphenyl)furan-2-carbaldehyde 54**

Obtained as a light brown solid in 63% yield. ^{1}H-NMR (DMSO-d_6), δ: 6.87-6.90 (m, 2H), 7.06 (d, J= 3.8 Hz, 1H), 7.60 (d, J= 3.8 Hz, 1H), 7.69-7.72 (m, 2H), 9.53 (s, 1H), 10.02 (bs, 1H). ^{13}C-NMR (DMSO-d_6), δ: 106.2, 115.7, 116.0, 119.4, 126.6, 150.6, 158.7, 158.9, 176.7.

13. Synthesis of 4-nitrophenyl benzoate 58

Benzoyl chloride **56** (6.5 mmol, 0.9 eq.) was added dropwise at 0 °C to a stirred solution of 4-nitrophenol **57** (7.2 mmol, 1 eq.) and pyridine (10.8 mmol, 1.5 eq.) in anhydrous DCM (36 mL). The reaction mixture was stirred for 20 hours. Water (20 mL) was then added, the organic phase was separated and washed with 2N aqueous HCl (20 mL) and brine (15 mL), dried over Na_2SO_4 and concentrated under *vacuum* to give the title compound as an off-white solid in quantitative yield. ^{1}H-NMR ($CDCl_3$), δ: 7.41-7.44 (m, 2H), 7.52-7.56 (m, 2H), 7.66-7.70 (m, 1H), 8.19-8.22 (m, 2H), 8.31-8.34 (m, 2H). ^{13}C-NMR ($CDCl_3$), δ: 122.7, 125.4, 128.6, 128.9, 130.4, 134.3, 145.5, 155.8, 164.3.

14. Synthesis of 4-aminophenyl benzoate 59

10% wet Pd/C (10% w/w) was added to a solution of 4-nitrophenyl benzoate **58** (28.8 mmol, 1 eq.) in anhydrous THF (196 mL). The reaction mixture was stirred under a hydrogen atmosphere for 24 hours. The catalyst Pd/C was removed by filtration through a celite pad, and the filtrate was concentrated under *vacuum* to give the title compound as a light brown solid in 97% yield. ^{1}H-NMR ($CDCl_3$), δ: 3.78 (bs, 2H), 6.70-6.73 (m, 2H), 6.99-7.02 (m, 2H), 7.48-7.52 (m, 2H), 7.60-7.64 (m, 1H), 8.18-8.20 (m, 2H). ^{13}C-NMR ($CDCl_3$), δ: 116.0, 122.6, 128.8, 130.4, 133.7, 143.4, 144.5, 166.0.

15. Synthesis of 4-hydrazineylphenyl benzoate 60

4-Aminophenyl benzoate **59** (13.8 mmol, 1 eq.) was dissolved in concentrated HCl (15 mL) and cooled to 0 °C. A solution of $NaNO_2$ (14.7 mmol, 1.1 eq.) in 15 mL of deionised water was added over 15 minutes. The solution was stirred for further 2 minutes at 0 °C, after which a solution of $SnCl_2$ (30.0 mmol, 2.2 eq.) in concentrated HCl (15 mL) was added dropwise. A precipitate formed. The reaction was stirred for 3 hours at 0 °C, then the solid was collected by filtration and washed with ice-cold concentrated HCl. The residue was purified by trituration in EtOH to give the title compound as an off-white solid in 77% yield. ^{1}H-NMR (DMSO-d_6), δ: 3.41 (bs, 1H), 7.05-7.08 (m, 1H), 7.27-7.32 (m, 2H), 7.21-7.24 (m, 1H), 7.59-7.63 (m, 2H), 7.73-7.78 (m, 1H), 8.11-8.15 (m, 2H), 10.06 (bs, 2H). ^{13}C-NMR (DMSO-d_6), δ: 115.3, 122.2, 128.8, 129.6, 133.8, 143.6, 144.7, 164.7.

16. Synthesis of 4-(2-(3-ethoxy-3-oxopropanoyl)hydrazineyl)phenyl benzoate 61

4-Hydrazineylphenyl benzoate **60** (8.6 mmol, 1.5 eq.) was dissolved in anhydrous THF (10 mL) under nitrogen atmosphere and Et_3N (5.7 mmol, 1 eq.) was added. The mixture was cooled to -10 °C and a solution of ethyl malonyl chloride (5.7 mmol, 1 eq.) in anhydrous THF (20 mL) was added dropwise. The reaction mixture was allowed to warm to room temperature and stirred for 3 hours. The reaction was concentrated under *vacuum* and the residue obtained was partitioned between EtOAc (3x 70 mL)

and water (20 mL). The combined organic layers were washed with brine (10 mL), dried over Na_2SO_4 and evaporated under *vacuum*. The residue was purified by automated flash column chromatography eluting with *n*-hexane/EtOAc 100:0 v/v increasing to *n*-hexane/EtOAc 0:100 v/v in 10 CV to give the title compound as an orange solid in 56% yield. ^{1}H-NMR (DMSO-d$_6$), δ: 1.22 (t, J= 7.1 Hz, 3H), 3.48 (s, 2H), 4.14 (q, J= 7.1 Hz, 2H), 7.23-7.27 (m, 2H), 7.60-7.67 (m, 4H), 7.74-7.76 (m, 1H), 8.12-8.15 (m, 2H), 10.30 (bs, 1H), 12.65 (bs, 1H). ^{13}C-NMR (DMSO-d$_6$), δ: 13.8, 43.4, 60.5, 119.9, 122.0, 128.7, 128.8, 129.6, 133.9, 136.5, 145.9, 163.9, 164.5, 167.4.

17. Synthesis of 1-(4-hydroxyphenyl)pyrazolidine-3,5-dione 62

4-(2-(3-Ethoxy-3-oxopropanoyl)hydrazineyl)phenyl benzoate **61** (1.2 mmol, 1 eq.) was dissolved in ethanol (2.5 mL) and an ethanolic 1M NaOH solution (2.5 mL) was added. The reaction mixture was stirred at room temperature for 30 minutes., then acidified by addition of a 1M aqueous HCl solution. The solvent was removed under *vacuum* and the residue was then extracted with EtOAc (3x 10 mL). The combined organic layers were dried over Na_2SO_4 and concentrated under *vacuum*. The residue was purified by automated flash column chromatography eluting with DCM/MeOH 100:0 v/v increasing to DCM/MeOH 90:10 v/v in 12 CV to give the title compound as an off-white solid in 73% yield. ^{1}H-NMR (DMSO-d$_6$), δ: 3.29 (s, 2H), 6.68-6.71 (m, 2H), 7.34-7.37 (m, 2H), 9.20 (bs, 1H), 9.86 (s, 1H). ^{13}C-NMR (DMSO-d$_6$), δ: 44.2, 115.5, 121.2, 131.1, 153.8, 164.2, 169.8.

Preparation and characterisation of final products

1. General procedure for the preparation of 2-((3-(2-hydroxyphenyl)-1H-1,2,4-triazol-5-yl)thio)-*N*-(aryl)acetamides 1 and 1a-d

A mixture of 5-(2-hydroxyphenyl)-2,4-dihydro-3H-1,2,4-triazole-3-thione **10** (1 mmol, 1 eq.), the appropriate 2-chloro-*N*-(aryl)acetamide **12a-e** (1 mmol, 1 eq) and pyridine (1 mmol, 1 eq.) was refluxed in EtOH (3 mL/mmol) overnight. The solvent was then removed under reduced pressure, and the residue was partitioned between H_2O (15 mL) and EtOAc (20 mL). The organic layer was dried over Na_2SO_4 and concentrated *under vacuum*. The crude residue was purified by recrystallisation from EtOH.

• *N*-(2,4-Dimethylphenyl)-2-((5-(2-hydroxyphenyl)-1*H*-1,2,4-triazol-3-yl)thio)acetamide **1**

Obtained as a white solid in 53% yield. ^{1}H-NMR ($CDCl_3$), δ: 2.09 (s, 3H), 2.24 (s, 3H), 4.02 (s, 2H) 6.92-6.96 (m, 3), 7.07 (d, J= 8.4 Hz, 1H), 7.34-7.38 (m, 1H), 7.52 (d, J= 8.4 Hz, 1H), 7.75-7.80 (m, 1H), 8.49 (bs, 1H), 10.57 (bs, 1H), 12.36 (bs, 1H). ^{13}C-NMR ($CDCl_3$), δ: 18.0, 20.9, 36.2, 117.2, 119.8, 125.3, 126.9, 127.9, 131.2, 132.3, 131.9, 133.8, 134.8, 156.2, 166.7. UPLC-MS (Method B): t_R 2.00 min, MS [ESI, m/z]: 355.2 $[M+H]^+$. HRMS calculated for $C_{18}H_{19}N_4O_2S^+$: 355.1223; found 355.1228.

• 2-((5-(2-Hydroxyphenyl)-1*H*-1,2,4-triazol-3-yl)thio)-*N*-(*o*-tolyl)acetamide **1a**

Obtained as a white solid in 56% yield. ^{1}H-NMR (DMSO-d_6), δ: 2.16 (s, 3H), 4.13 (s, 2H) 6.94-6.97 (m, 1H), 6.99-7.02 (m, 1H), 7.05-7.09 (m, 1H), 7.13-7.17 (m, 1H), 7.18-7.20 (m, 1H), 7.32-7.35 (m, 1H), 7.41-7.44 (m, 1H), 7.92 (dd, J_1= 7.8 Hz, J_2= 1.4 Hz, 1H) 9.61 (bs, 1H), 10.98 (bs, 1H), 14.15 (bs, 1H). ^{13}C-NMR (DMSO-d_6), δ: 18.1, 36.2, 117.0, 120.0, 125.1, 125.8, 126.5, 127.8, 130.7, 132.1, 132.0, 136.5, 156.1, 166.83. UPLC-MS (Method B): t_R 1.94 min, MS [ESI, m/z]: 341.2 $[M+H]^+$. HRMS calculated for $C_{17}H_{17}N_4O_2S^+$: 341.1067; found 341.1071.

• 2-((5-(2-Hydroxyphenyl)-1*H*-1,2,4-triazol-3-yl)thio)-*N*-(*p*-tolyl)acetamide **1b**

Obtained as a white solid in 54% yield. ^{1}H-NMR (DMSO-d_6), δ: 2.25 (s, 3H), 4.10 (s, 2H) 6.93-6.96 (m, 1H), 6.99-7.02 (m, 1H), 7.05-7.09 (m, 1H), 7.13-7.17 (m, 1H), 6.97-7.00 (m, 1H), 7.11 (d, J= 8.1 Hz, 2H), 7.31-7.35 (m, 1H), 7.46 (d, J= 8.1 Hz, 2H), 7.90 (dd, J_1= 7.8 Hz, J_2= 1.6 Hz, 1H) 10.21 (bs, 1H), 10.93 (bs, 1H), 14.14 (bs, 1H). ^{13}C-NMR (DMSO-d_6), δ: 20.9, 36.7, 117.1, 119.6, 119.9, 127.8, 129.6, 132.1, 121.8, 132.8, 136.8, 156.0, 162.7, 166.3, 176.6. UPLC-MS (Method C): t_R 1.74 min, MS [ESI, m/z]: 341.1 $[M+H]^+$. HRMS calculated for $C_{17}H_{17}N_4O_2S^+$: 341.1067; found 341.1061.

• 2-((5-(2-Hydroxyphenyl)-1*H*-1,2,4-triazol-3-yl)thio)-*N*-(4-(trifluoromethyl)phenyl)acetamide **1c**

Obtained as a white solid in 71% yield. ^{1}H-NMR (DMSO-d_6), δ: 4.16 (s, 2H) 6.92-6.95 (m, 1H), 6.98-7.00 (m, 1H), 7.31-7.34 (m, 1H), 7.68 (d, J= 8.5 Hz, 2H), 7.80 (d, J= 8.5 Hz, 2H), 7.88 (dd, J_1= 7.8 Hz, J_2= 1.6 Hz, 1H) 10.67 (bs, 1H), 10.92 (bs, 1H), 14.13 (bs, 1H). ^{19}F-NMR (DMSO-d_6), δ: -60.33. ^{13}C-NMR (DMSO-d_6), δ: 36.7, 123.7, 124.0, 125.8, 128.0, 142.9, 156.0, 117.1, 119.5, 119.9, 126.5, 127.8, 132.1, 156.0, 167.3. UPLC-

MS (Method B): t_R 2.12 min, MS [ESI, m/z]: 395.2 $[M+H]^+$. HRMS calculated for $C_{17}H_{14}F_3N_4O_2S^+$: 395.0784; found 395.0788.

• *N*-(3-Chloro-4-methylphenyl)-2-((5-(2-hydroxyphenyl)-1*H*-1,2,4-triazol-3-yl)thio)acetamide **1d**

Obtained as a white solid in 67% yield. ^{1}H-NMR (DMSO-d_6), δ: 2.26 (s, 3H), 4.10 (s, 2H) 6.92-6.95 (m, 1H), 6.98-7.00 (m, 1H), 7.27 (d, J= 8.3 Hz, 1H), 7.31-7.37 (m, 2H), 7.78 (d, J= 1.9 Hz, 1H), 7.89 (dd, J_1= 8.3 Hz, J_2= 1.9 Hz, 1H), 10.40 (bs, 1H), 10.95 (bs, 1H), 14.15 (bs, 1H). ^{13}C-NMR (DMSO-d_6), δ: 19.4, 36.6, 117.1, 118.2, 119.5, 119.9, 127.8, 131.7, 132.1, 130.5, 133.4, 138.5, 156.0, 166.8. UPLC-MS (Method B): t_R 2.13 min, MS [ESI, m/z]: 375.2, 377.2 $[M+H]^+$. HRMS calculated for $C_{17}H_{16}ClN_4O_2S^+$: 375.0677; found 375.0682.

2. Synthesis of *N*-(2,4-dimethylphenyl)-2-((5-(2-hydroxyphenyl)-1H-1,2,4-triazol-3-yl)sulfinyl)acetamide 1e

m-CPBA (0.05 g, 0.29 mmol) dissolved in dry DCM (5 mL) was added portion-wise to a cooled solution of *N*-(2,4-dimethylphenyl)-2-((3-(2-hydroxyphenyl)-1H-1,2,4-triazol-5-yl)thio)acetamide **1** (0.07 g, 0.19 mmol), under vigorous stirring. The reaction was stirred for 5 days at room temperature, then quenched with saturated Na_2SO_3 and extracted with DCM (3x 20 mL). The organic layer was dried over Na_2SO_4 and concentrated under reduced pressure. The crude residue was purified by recrystallisation from EtOH to afford the title compound as a grey solid in 34% yield. ^{1}H-NMR (DMSO-d_6), δ: 2.10 (s, 2H), 2.24 (s, 3H), 4.35 (d, J= 13.7 Hz, 1H), 4.60 (d, J= 13.7 Hz, 1H), 6.95-6.97 (m, 1H), 6.98-7.01 (m, 2H), 7.05 (dd, J_1= 8.2 Hz, J_2= 0.7 Hz, 1H), 7.23 (d, J= 8.2 Hz, 1H), 7.36-7.39 (m, 1H), 8.0 (dd, J_1= 7.8 Hz, J_2= 1.5 Hz, 1H), 9.77 (bs, 1H), 11.17 (bs, 1H), 14.24 (bs, 1H). ^{13}C-NMR (DMSO-d_6), δ: 18.2, 20.7, 58.9, 116.8, 120.1, 125.4, 127.0, 128.9, 131.3, 132.6, 113.2, 132.3, 133.2, 135.4, 155.36, 155.9, 163.3, 163.6. UPLC-MS (Method B): t_R 1.76 min, MS [ESI, m/z]: 371.3 $[M+H]^+$. HRMS calculated for $C_{18}H_{19}N_4O_3S^+$: 371.1172; found 371.1165.

3. Synthesis of *N*-(2,4-dimethylphenyl)-2-((5-(2-hydroxyphenyl)-1H-1,2,4-triazol-3-yl)sulfonyl)acetamide 1f

A mixture of *N*-(2,4-dimethylphenyl)-2-((3-(2-hydroxyphenyl)-1H-1,2,4-triazol-5-yl)thio) acetamide **1** (0.16 g, 0.45 mmol), niobium pentaethoxide (0.003 g, 0.009 mmol) and 30% hydrogen peroxide (0.06 g, 1.80 mmol) in MeOH (2 mL) was stirred at 45 ^{o}C for 2 hours. The reaction was quenched with saturated Na_2SO_3 and extracted with EtOAc (3x 20 mL). The organic layer was dried over Na_2SO_4 and concentrated under reduced pressure to give the pure title compound as an off-white solid in 87% yield. ^{1}H-NMR (DMSO-d_6), δ: 2.13 (s, 3H), 2.23 (s, 3H), 4.64 (s, 2H), 6.93-6.99 (m, 2H), 7.00-7.05 (m, 2H), 7.05 (dd, J_1= 8.2 Hz, J_2= 0.7 Hz, 1H), 7.22 (d, J= 7.8 Hz, 1H), 7.34-7.38 (m, 1H), 7.95 (d, J= 7.8 Hz, 1H), 9.67 (bs, 1H), 12.74 (bs, 1H). ^{13}C-NMR (DMSO-d_6), δ: 18.3, 20.9, 60.9, 116.8, 120.0, 125.0, 126.8, 129.0, 131.2, 132.5,

113.5, 132.0, 132.5, 135.2, 156.3, 159.4, 160.1. UPLC-MS (Method B): t_R 1.94 min, MS [ESI, m/z]: 387.3 $[M+H]^+$. HRMS calculated for $C_{18}H_{19}N_4O_4S^+$: 387.1122; found 387.1126.

4. General procedure for the preparation of *N*-(3-(1,3-dioxoisoindoline)phenyl)benzamides 2, 2a-b and 2d-e

The appropriate (3-amino)-1,3-dioxoisoindoline **23-26** (1 mmol, 1 eq.) was treated with differently substituted benzoyl chlorides **28a-c** (1.2 mmol, 1.2 eq.) in dry THF (5 ml) under a N_2 atmosphere in the presence of pyridine (1.4 mmol, 1.4 eq.). The reaction was stirred at room temperature overnight. The solvent was removed under *vacuum*, and the residue was taken up in EtOAc (25 mL). The organic layer was washed with water (20 mL), 1M HCl solution (20 mL) and brine (15 mL), then dried over MgSO4 and concentrated under *vacuum*. The crude residue was purified by flash column chromatography.

• 2-(3-Benzamido-4-methoxyphenyl)-1,3-dioxoisoindoline-5-carboxylic acid **2**

Purified by automated flash column chromatography eluting with DCM:MeOH 100:0 v/v increasing to DCM:MeOH 90:10 v/v in 15 CV. Obtained as a yellow solid in 41% yield. ^{1}H-NMR (DMSO-d_6), δ: 3.96 (s, 3H), 7.25 (d, J= 8.8 Hz, 1H), 7.29 (dd, J_1= 8.8 Hz, J_2= 2.3 Hz, 1H), 7.52-7.56 (m, 2H), 7.59-7.63 (m, 1H), 7.91 (d, J= 2.3 Hz, 1H), 7.95-7.99 (m, 2H), 8.07 (d, J= 7.7 Hz, 1H), 8.31 (s, 1H), 8.41 (dd, J_1= 7.7 Hz, J_2= 1.3 Hz, 1H), 9.57 (bs, 1H), 13.78 (bs, 1H). ^{13}C-NMR (DMSO-d_6), δ: 56.7, 111.9, 123.5, 123.7, 123.9, 125.4, 128.0, 129.0, 132.2, 135.6, 124.4, 127.4, 132.0, 134.7, 151.6, 165.5, 167.2, 167.4, 167.5. UPLC-MS (Method B): t_R 1.97 min, MS [ESI, m/z]: 417.3 $[M+H]^+$. HRMS calculated for $C_{23}H_{17}N_2O_6^+$: 417.1081; found 417.1070.

• 2-(3-Benzamidophenyl)-1,3-dioxoisoindoline-5-carboxylic acid **2a**

Purified by automated flash column chromatography eluting with DCM:MeOH 100:0 v/v increasing to DCM:MeOH 90:10 v/v in 10 CV. Obtained as a white solid in 36% yield. ^{1}H-NMR (DMSO-d_6), δ: 7.20-7.22 (m, 1H), 7.50-7.53 (m, 1H), 7.54-7.56 (m, 2H), 7.59-7.62 (m, 1H), 7.82-7.84 (m, 1H) 7.96-7.98 (m, 1H), 7.99-8.00 (m, 2H), 8.09 (dd, J_1= 7.6 Hz, J_2= 0.5 Hz, 1H), 8.32-8.33 (m, 1H), 8.43 (dd, J_1= 8.0 Hz, J_2= 1.3 Hz, 1H) 10.48 (bs, 1H), 13.72 (bs, 1H). ^{13}C-NMR (DMSO-d_6), δ: 119.5, 120.5, 123.0, 123.8, 124.2, 128.2, 128.8, 129.4, 132.2, 135.9, 132.4, 132.4, 135.1, 135.9, 140.2, 166.1, 166.3, 166.7, 166.8. UPLC-MS (Method B): t_R 1.88 min, MS [ESI, m/z]: 387.2 $[M+H]^+$. HRMS calculated for $C_{22}H_{15}N_2O_5^+$: 387.0975; found 387.0989.

• 2-(4-Methoxy-3-(4-methylbenzamido)phenyl)-1,3-dioxoisoindoline-5-carboxylic acid **2b**

Purified by automated flash column chromatography eluting with DCM:MeOH 100:0 v/v increasing to DCM:MeOH 90:10 v/v in 10 CV. Obtained as a pale yellow solid in 58% yield. ^{1}H-NMR (DMSO-d_6), δ: 2.39 (s, 3H), 3.91 (s, 3H), 7.24 (d, J= 8.7 Hz, 1H), 7.27 (dd, J_1= 8.7 Hz, J_2= 2.2 Hz, 1H), 7.33 (d, J= 7.1 Hz, 2H), 7.87 (d, J= 7.1 Hz, 2H), 7.92 (d, J= 2.2 Hz, 1H), 8.06 (d, J= 7.7 Hz, 1H), 8.31 (s, 1H), 8.41 (dd, J_1= 7.7Hz, J_2= 1.3 Hz, 1H), 9.47 (bs, 1H), 13.80 (bs, 1H). ^{13}C-NMR (DMSO-d_6), δ: 21.5, 56.6, 111.9, 123.5, 123.7, 124.1, 125.2, 128.0, 129.5, 135.8, 124.3, 127.6, 131.9, 132.5, 135.3, 142.2, 151.6, 165.3, 166.4, 167.0, 167.0. UPLC-

MS (Method B): t_R 2.11 min, MS [ESI, m/z]: 431.2 $[M+H]^+$. HRMS calculated for $C_{24}H_{19}N_2O_6^+$: 431.1238; found 431.1232.

• *N*-(5-(1,3-Dioxoisoindolin-2-yl)-2-methoxyphenyl)benzamide **2d**

Purified by automated flash column chromatography eluting with EtOAc:MeOH 100:0 v/v increasing to EtOAc:MeOH 90:10 v/v in 10 CV. Obtained as a white solid in 43% yield. ^{1}H-NMR (DMSO-d_6), δ: 3.91 (s, 3H), 7.24 (d, J= 8.7 Hz, 1H), 7.28 (dd, J_1= 8.7 Hz, J_2= 2.3 Hz, 1H), 7.52-7.55 (m, 2H), 7.59-7.62 (m, 1H), 7.89 (d, J= 2.3 Hz, 1H), 7.90-7.92 (m, 2H), 7.95-7.96 (m, 1H), 7-97-7.99 (m, 3H), 9.57 (bs, 1H). ^{13}C-NMR (DMSO-d_6), δ: 56.7, 111.9, 123.8, 123.8, 125.4, 127.9, 129.0, 132.2, 135.0, 124.5, 127.5, 132.0, 134.7, 151.7, 165.5, 167.2. UPLC-MS (Method B): t_R 2.14 min, MS [ESI, m/z]: 373.2 $[M+H]^+$. HRMS calculated for $C_{22}H_{17}N_2O_4^+$: 373.1183; found 373.1189.

• *N*-(3-(1,3-Dioxoisoindolin-2-yl)phenyl)benzamide **2e**

Purified by automated flash column chromatography eluting with *n*-hexane:EtOAc 100:0 v/v increasing to *n*-hexane:EtOAc 0:100 v/v in 10 CV. Obtained as a pale yellow solid in 59% yield. ^{1}H-NMR (DMSO-d_6), δ: 7.18-7.22 (m, 1H), 7.49-7.53 (m, 1H), 7.54-7.56 (m, 2H), 7.59-7.62 (m, 1H), 7.81-7.84 (m, 1H) 7.91-7.93 (m, 2H), 7.94-7.95 (m, 1H), 7.97-8.00 (m, 4H), 10.47 (bs, 1H). ^{13}C-NMR (DMSO-d_6), δ: 119.6, 120.4, 123.1, 123.9, 128.1, 128.8, 129.4, 132.1, 135.2, 132.0, 132.5, 135.1, 140.1, 166.1, 167.4. UPLC-MS (Method B): t_R 2.05 min, MS [ESI, m/z]: 343.2 $[M+H]^+$. HRMS calculated for $C_{21}H_{15}N_2O_3^+$: 343.1077; found 343.1069.

5. General procedure for the preparation of *N*-(3-(1,3-dioxoisoindoline)phenyl)benzamides 2c and 2f

A solution of the differently substituted benzoyl chloride **28a-b** (1.1 mmol, 1.1 eq.) in anhydrous DCM (7 mL) was added to the appropriate (3-amino)-1,3-dioxoisoindoline **23** or **27** (1 mmol, 1 eq.) under a N_2 atmosphere. The mixture was stirred at room temperature for 3 hours, then Et_3N (1.3 mmol, 1.3 eq.) was added, and stirring was continued overnight. in the presence of pyridine (1.4 mmol, 1.4 eq.). The reaction was stirred at room temperature overnight. The solvent was removed under *vacuum*, and the residue was taken up in EtOAc (25 mL). The organic layer was washed with water (20 mL), 1M HCl solution (20 mL) and brine (15 mL), then dried over MgSO4 and concentrated under *vacuum*. The crude residue was purified by flash column chromatography.

• 2-(4-Methoxy-3-(4-methoxybenzamido)phenyl)-1,3-dioxoisoindoline-5-carboxylic acid **2c**

Purified by automated flash column chromatography eluting with DCM:MeOH 100:0 v/v increasing to DCM:MeOH 90:10 v/v in 10 CV. Obtained as a yellow solid in 37% yield. ^{1}H-NMR (DMSO-d_6), δ: 3.84 (s, 3H), 3.91 (s, 3H), 7.11 (d, J= 8.7 Hz, 2H), 7.21-7.27 (m, 2H), 7.89 (dd, J_1= 8.1 Hz, J_2= 1.7 Hz, 1H), 7.92-7.98 (m, 3H), 8.35 (s, 1H), 8.38 (d, J=8.1 Hz, 1H), 9.41 (bs, 1H), 13.76 (bs, 1H). ^{13}C-NMR (DMSO-d_6), δ: 55.9, 56.6, 111.8, 114.2, 123.4, 123.5, 123.9, 125.1, 129.9, 135.5, 124.5, 126.8, 127.7, 131.9, 132.7, 133.0, 151.5,

162.4, 164.9, 167.5, 167.6. UPLC-MS (Method B): t_R 2.01 min, MS [ESI, m/z]: 447.2 $[M+H]^+$. HRMS calculated for $C_{24}H_{19}N_2O_7^+$: 447.1187; found 447.1181.

• *N*-(5-(5-Hydroxy-1,3-dioxoisoindolin-2-yl)-2-methoxyphenyl)benzamide **2f**

Purified by automated flash column chromatography eluting with DCM:MeOH 100:0 v/v increasing to DCM:MeOH 95:5 v/v in 10 CV. Obtained as a yellow solid in 57% yield. ^{1}H-NMR (DMSO-d_6), δ: 3.91 (s, 3H), 7.19 (dd, J_1= 8.2 Hz, J_2= 2.2 Hz, 1H), 7.22-7.23 (m, 3H), 7.52-7.56 (m, 2H), 7.59-7.62 (m, 1H), 7.79 (dd, J_1= 8.2 Hz, J_2= 0.4 Hz, 1H), 7.85 (m, 1H), 7.97-7.98 (m, 2H), 9.56 (bs, 1H), 11.06 (bs, 1H). ^{13}C-NMR (DMSO-d_6), δ: 56.3, 110.4, 111.9, 121.2, 122.1, 123.7, 124.7, 126.1, 127.5, 127.9, 129.0, 129.7, 132.3, 134.7, 134.8, 151.5, 163.9, 165.5, 167.5. UPLC-MS (Method B): t_R 2.04 min, MS [ESI, m/z]: 389.2 $[M+H]^+$. HRMS calculated for $C_{22}H_{17}N_2O_5^+$: 389.1132; found 389.1120.

6. Synthesis of methyl 2-(3-benzamido-4-methoxyphenyl)-1,3-dioxoisoindoline-5-carboxylate 2g

A mixture of the carboxylic acid **2** (1 mmol, 1 eq.), sulfuric acid (1 mL) and methanol (10 mL) was heated under reflux overnight. The solvent was evaporated under *vacuum*, water (10 mL) was added and the mixture was neutralized to pH 6-7 using solid sodium bicarbonate. The water layer was extracted with DCM (3x 20 mL), and the combined organic extracts were dried over $MgSO_4$ and concentrated under *vacuum*. The crude residue was purified by flash column chromatography eluting with DCM:MeOH 100:0 v/v increasing to DCM:MeOH 90:10 v/v in 10 CV. The title compound was obtained as a yellow oil in 89% yield. ^{1}H-NMR ($CDCl_3$), δ: 3.91 (s, 3H), 3.93 (s, 3H), 6.96 (d, J=8.7 Hz, 1H), 7.05 (dd, J_1= 8.7 Hz, J_2= 2.5 Hz, 1H), 7.41-7.44 (m, 2H), 7.46-7.50 (m, 1H), 7.80-7.82 (m, 2H), 7.95 (dd, J_1= 7.7 Hz, J_2= 0.7 Hz, 1H), 8.40 (dd, J_1= 7.7 Hz, J_2= 1.4 Hz, 1H), 8.51-8.51 (m, 1H), 8.53 (s, 1H), 8.60 (d, J= 2.5 Hz, 1H). ^{13}C-NMR ($CDCl_3$), δ: 52.9, 56.2, 110.1, 118.4, 122.3, 123.7, 124.8, 127.1, 128.8, 131.9, 135.7, 123.7, 128.5, 132.0, 134.9, 135.2, 135.9, 147.9, 165.2, 166.5, 166.5. UPLC-MS (Method B): t_R 2.23 min, MS [ESI, m/z]: 431.3 $[M+H]^+$. HRMS calculated for $C_{24}H_{19}N_2O_6^+$: 431.1238; found 431.1244.

7. Synthesis of 2-(3-benzamido-4-methoxyphenyl)-1,3-dioxoisoindolin-5-yl dihydrogen phosphate (* Et_3N) 2h

N-(5-(5-Hydroxy-1,3-dioxoisoindolin-2-yl)-2-methoxyphenyl)benzamide **2f** (0.25 mmol, 1 eq.) was dissolved in anhydrous DCM (10 mL) under a N_2 atmosphere. Et_3N (0.7 mmol, 2.9 eq.) was added at room temperature, and the mixture was cooled to 0 °C, followed by the addition of diethyl chlorophosphate (0.29 mmol, 1.2 eq.). The reaction mixture was stirred at 0 °C for 2 hours, then the solvent was evaporated under reduced pressure. The crude residue was purified by automated flash column chromatography eluting with *n*-hexane:EtOAc 85:15 v/v increasing to *n*-hexane:EtOAc 0:100 v/v in 10 CV, affording intermediate 2-(3-benzamido-4-methoxyphenyl)-1,3-dioxoisoindolin-5-yl diethyl phosphate as a yellow solid in 65% yield. ^{1}H-NMR ($CDCl_3$), δ: 1.40 (td, J_1= 14.2 Hz, J_2= 7.1 Hz,

1H), 3.99 (s, 3H), 4.25-4.32 (m, 4H), 7.03 (d, J= 8.7 Hz, 1H), 7.11 (dd, J_1= 8.6 Hz, J_2= 2.5 Hz, 1H), 7.49-7.52 (m, 2H), 7.54-7.58 (m, 1H), 7.62-7.64 (m, 1H), 7.76-7.77 (m, 1H), 7.88-7.89 (m, 2H), 7.94 (dt, J_1= 8.1 Hz, J_2= 0.5 Hz, 1H), 8.60 (bs, 1H), 8.66 (d, J= 2.5 Hz, 1H). ^{13}C-NMR ($CDCl_3$), δ: 16.2, 16.3, 56.3, 64.3, 65.4, 110.3, 115.7, 118.6, 122.5, 124.7, 125.7, 125.9, 127.2, 128.1, 128.6, 128.9, 131.9, 134.2, 135.2, 147.9, 155.7 (d, J_P= 6.6 Hz), 165.3, 166.5 (d, J_P= 27.6 Hz). ^{31}P-NMR ($CDCl_3$), δ: -6.97.

2-(3-Benzamido-4-methoxyphenyl)-1,3-dioxoisoindolin-5-yl diethyl phosphate (0.14 mmol, 1 eq.) was dissolved in anhydrous DCM (4 mL) under a N_2 atmosphere, and the mixture was cooled to 0 °C. Pyridine (0.55 mmol, 4 eq.) was added, followed by TMSBr (0.7 mmol, 5 eq.). The reaction mixture was stirred at room temperature overnight. After this time, further pyridine (0.55 mmol, 4 eq.) and TMSBr (0.7 mmol, 5 eq.) were added at 0 °C, and the reaction mixture was stirred at room temperature for further 48 hours. MeOH (2 mL) was then added at 0 °C, and the reaction mixture was stirred at room temperature for 15 minutes. The solvents were evaporated at reduced pressure, and the crude residue was purified by automated flash column chromatography eluting with DCM:MeOH:Et_3N 96:0:4 v/v increasing to DCM:MeOH:Et_3N 63:30:7 v/v in 15 CV, affording the title compound as a yellow solid in 32% yield (triethylamine salt). ^{1}H-NMR (DMSO-d_6), δ: 1.14-1.25 (m, 9H), 2.91-3.05 (m, 6H), 3.91 (s, 3H), 7.20-7.30 (m, 2H), 7.46-7.49 (m, 1H), 7.51-7.62 (m, 3H), 7.82-7.84 (m, 3H), 7.96-7.98 (m, 2H), 9.57 (bs, 1H). ^{13}C-NMR (DMSO-d_6), δ: 45.9, 52.5, 56.6, 111.9, 114.7, 123.8, 124.0, 124.7, 125.1, 125.4, 127.5, 127.9, 128.9, 129.0, 132.2, 133.8, 134.8, 151.6, 160.8 (d, J_P= 7.5 Hz), 165.5, 167.4 (d, J_P= 8.4 Hz). ^{31}P-NMR (DMSO-d_6), δ: -5.31. UPLC-MS (Method B): t_R 1.74 min, MS [ESI, m/z]: 469.1 $[M+H]^+$. HRMS calculated for $C_{22}H_{18}N_2O_8P^+$: 469.0795; found 469.0784.

8. General procedure for the preparation of benzohydrazone 3a-b

The appropriate benzaldehyde of acetophenone **38-41** (1.1 mmol, 1.1 eq.) was added to a solution of the differently substituted benzohydrazide **8**, **35-37** (1 mmol, 1 eq.) in EtOH (7 mL). The mixture was stirred at reflux temperature overnight, then it was cooled to room temperature, and the precipitate formed was collected by filtration and washed with cold EtOH. The crude residue was purified by recrystallization from EtOH.

• 2-(Benzyloxy)-*N'*-(2,4-dihydroxybenzylidene)benzohydrazide **3a**

Obtained as an off-white solid in 47% yield. ^{1}H-NMR (DMSO-d_6), δ: 5.24 (s, 2H), 6.31 (d, J= 2.3 Hz, 1H), 6.35 (dd, J_1= 8.4 Hz, J_2= 2.3 Hz, 1H), 7.08-7.10 (m, 1H), 7.23-7.26 (m, 2H), 7.30-7.34 (m 1H), 7.37-7.40 (m, 2H), 7.48-7.50 (m, 1H), 7.51-7.54 (m, 2H), 7.63 (dd, J_1= 7.5 Hz, J_2= 1.7 Hz, 1H), 8.22 (s, 1H), 9.96 (bs, 1H), 11.32 (bs, 1H), 11.55 (bs, 1H). ^{13}C-NMR (DMSO-d_6), δ: 70.3, 103.1, 108.2, 110.9, 113.8, 121.3, 124.1, 127.6, 128.0, 128.4, 128.7, 128.9, 130.4, 131.6, 132.6, 137.2, 148.6, 156.2, 159.8, 161.1, 162.1. UPLC-MS (Method B): t_R 2.10 min, MS [ESI, m/z]: 363.3 $[M+H]^+$. HRMS calculated for $C_{21}H_{19}N_2O_4^+$: 363.1339; found 363.1345.

• *N'*-(4-(Benzyloxy)-2-hydroxybenzylidene)-2-hydroxybenzohydrazide **3b**

Obtained as a white solid in 57% yield. ^{1}H-NMR (DMSO-d_6), δ: 5.14 (s, 2H), 6.59 (d, J= 2.4 Hz, 1H), 6.62 (dd, J_1= 8.5 Hz, J_2= 2.4 Hz, 1H), 6.95-6.99 (m, 2H), 7.33-7.36 (m, 1H), 7.39-7.42 (m 2H), 745-7.47 (m, 4H), 7.88 (dd, J_1= 7.9 Hz, J_2= 1.5 Hz, 1H), 8.59 (s, 1H), 11.53 (bs, 1H), 11.94 (bs, 1H), 11.98 (bs, 1H). ^{13}C-NMR (DMSO-d_6), δ: 69.8, 102.5, 107.7, 112.3, 116.0, 117.7, 119.4, 128.2, 128.4, 128.9, 128.9, 131.6, 134.2, 137.1, 149.9, 159.5, 159.9, 161.7, 164.7. UPLC-MS (Method B): t_R 2.02 min, MS [ESI, m/z]: 363.3 [M+H]$^+$. HRMS calculated for $C_{21}H_{19}N_2O_4^+$: 363.1339; found 363.13334.

9. General procedure for the preparation of benzohydrazone-phopshates 3c-f

The appropriate diethyl phosphate intermediate **46-49** (0.25 mmol, 1 eq.) was dissolved in anhydrous DCM (4 mL) at 0 °C; pyridine (1 mmol, 4 eq.) and bromotrimethylsilane (1.25 mmol, 5 eq.) were then added to the reaction. The mixture was stirred at room temperature overnight. MeOH (3 mL) was added, and the mixture was stirred at room temperature for further 15 minutes. The solvents were evaporated at reduced pressure, and the crude residue was purified by automated flash column chromatography eluting with DCM:MeOH:Et_3N 95:0:5 v/v increasing to DCM:MeOH:Et_3N 70:25:5 v/v in 15 CV, affording the title compound as a triethylamine salt.

• 4-(2-(1-(2-Fluorophenyl)ethylidene)hydrazine-1-carbonyl)phenyl dihydrogen phosphate **3c**

Obtained as a white solid in 54% yield. ^{1}H-NMR (DMSO-d_6), δ: 1.05-1.08 (m, 9H), 2.36 (s, 3H), 2.78-2.78 (m, 6H), 7.23-7.30 (m, 4H), 7.45-7.49 (m, 1H), 7.59-7.62 (m, 1H), 7.78-7.80 (m, 2H), 10.62 (bs, 1H). ^{13}C-NMR (DMSO-d_6), δ: 7.6, 9.9, 45.9, 52.4, 116.5, 116.6, 119.6, 124.9, 124.9, 126.9, 127.8, 127.9, 130.3, 159.4, 161.4. ^{31}P-NMR (DMSO-d_6), δ: -5.53. UPLC-MS (Method B): t_R 1.38 min, MS [ESI, m/z]: 353.2 [M+H]$^+$. HRMS calculated for $C_{15}H_{15}FN_2O_5P^+$: 353.0697; found 353.0699.

• 3-(2-(1-(2-Fluorophenyl)ethylidene)hydrazine-1-carbonyl)phenyl dihydrogen phosphate **3d**

Obtained as a white solid in 95% yield. ^{1}H-NMR (DMSO-d_6), δ: 1.13-1.15 (m, 9H), 2.36 (s, 3H), 2.93-2.97 (m, 6H), 7.27-7.35 (m, 4H), 7.44-7.48 (m, 2H), 7.61-7.62 (m, 1H), 10.81 (bs, 1H). ^{13}C-NMR (DMSO-d_6), δ: 9.1, 10.2, 45.7, 116.5, 116.6, 124.9, 124.9, 127.7, 127.8, 130.3, 130.3, 131.5, 131.5, 159.4, 161.4. ^{31}P-NMR (DMSO-d_6), δ: -5.09. UPLC-MS (Method B): t_R 1.42 min, MS [ESI, m/z]: 353.2 [M+H]$^+$. HRMS calculated for $C_{15}H_{15}FN_2O_5P^+$: 353.0697; found 353.0710.

• 2-(2-(1-(2-Fluorophenyl)ethylidene)hydrazine-1-carbonyl)phenyl dihydrogen phosphate **3e**

Obtained as a white solid in 63% yield. ^{1}H-NMR (DMSO-d_6), δ: 1.14-1.18 (m, 9H), 2.42 (s, 3H), 2.99-3.03 (m, 6H), 7.12-7.15 (m, 1H), 7.26-7.29 (m, 2H), 7.41-7.47 (m, 3H), 7.60-7.63 (m, 1H), 7.73-7.75 (m, 1H), 12.37 (bs, 1H). ^{13}C-NMR (DMSO-d_6), δ: 7.9, 9.1, 45.9, 52.4, 116.4, 116.6, 123.0, 124.8, 124.8, 126.5, 130.3, 130.4, 131.1, 132.1, 150.9, 159.5, 161.4, 163.0. ^{31}P-NMR (DMSO-d_6), δ: -3.98. UPLC-MS (Method B): t_R 1.39 min, MS [ESI, m/z]: 353.2 [M+H]$^+$. HRMS calculated for $C_{15}H_{15}FN_2O_5P^+$: 353.0697; found 353.0692.

• 2-(1-(2-Benzoylhydrazineylidene)ethyl)phenyl dihydrogen phosphate **3f**

Obtained as a white solid in 56% yield. ^1H-NMR (CD_3OD), δ: 1.17-1.20 (m, 9H), 2.31 (s, 3H), 3.04-3.09 (m, 6H), 7.00-7.62 (m, 8H), 7.76-7.83 (m, 1H). ^{13}C-NMR (CD_3OD), δ: 7.7, 38.8, 46.3, 67.7, 120.4, 121.4, 122.5, 123.6, 127.1, 127.5, 128.3, 128.4, 131.3, 131.8, 132.2, 132.9, 167.9. ^{31}P-NMR (CD_3OD), δ: -3.75. UPLC-MS (Method B): t_R 1.34 min, MS [ESI, m/z]: 335.1 $[M+H]^+$. HRMS calculated for $C_{15}H_{16}N_2O_5P^+$: 335.0791; found 335.0797.

10. General procedure for the preparation of thiazolidine-2,4-diones 4a-b

The appropriate phenyl)furan-2-carbaldehyde **53-54** (0.8 mmol, 1 eq.), 3-(2-morpholino-2-oxoethyl)thiazolidine-2,4-dione **55** (1.2 mmol, 1.5 eq.) and β-alanine (1.2 mmol, 1.5 eq.) were stirred at 100 °C in glacial acetic acid (8 mL) overnight. The reaction mixture was cooled to room temperature and then quenched by addition of distilled water (20 mL). The precipitate formed was collected by filtration and washed with distilled water, ice-cold methanol and *n*-hexane. The crude residue was purified by recrystallization from EtOH.

- 5-((5-4-Hydroxy-2-methylphenyl)furan-2-yl)methylene)-3-(2- morpholino-2-oxoethyl)thiazolidine-2,4-dione **4a**

Obtained as a brown solid in 29% yield. ^1H-NMR (DMSO-d_6), δ: 2.45 (s, 3H), 3.43-3.45 (m, 2H), 3.54-3.59 (m, 2H), 3.63-3.65 (m, 2H), 4.60 (s, 2H), 6.75-6.76 (m, 1H), 6.80 (dd, J_1= 8.5, J_2= 2.7, 1H), 6.88 (d, J= 3.7 Hz, 1H), 7.31 (dd, J_1= 3.7, J_2= 0.3, 1H), 7.62-7.63 (m, 1H), 7.80 (s, 1H), 9.88 (bs, 1H). ^{13}C-NMR (DMSO-d_6), δ: 21.8, 41.9, 42.4, 44.5, 65.9, 111.0, 113.7, 115.6, 118.1, 119.4, 119.5, 122.4, 128.8, 137.1, 147.4, 158.0, 158.2, 163.5, 165.1, 167.8. UPLC-MS (Method C): t_R 1.68 min, MS [ESI, m/z]: 429.3 $[M+H]^+$. HRMS calculated for $C_{21}H_{21}N_2O_6S$+: 429.1115; found 429.1103.

- 5-((5-(4-Hydroxyphenyl)furan-2-yl)methylene)-3-(2-morpholino-2-oxoethyl)thiazolidine-2,4-dione **4b**

Obtained as a brown solid in 67% yield. ^1H-NMR (DMSO-d_6), δ: 3.43-3.45 (m, 2H), 3.54-3.59 (m, 2H), 3.63-3.65 (m, 2H), 4.60 (s, 2H), 6.91-6.94 (m, 2H), 7.08 (d, J= 3.7 Hz, 1H), 7.27-7.28 (m, 1H), 7.68-7.71 (m, 2H), 7.78 (s, 1H), 10.00 (bs, 1H). ^{13}C-NMR (DMSO-d_6), δ: 41.2, 41.7, 43.8, 65.2, 106.8, 114.9, 115.5, 118.7, 119.2, 121.9, 125.6, 147.1, 157.6, 158.0, 162.8, 164.4, 167.3. UPLC-MS (Method C): t_R 1.85 min, MS [ESI, m/z]: 415.2 $[M+H]^+$. HRMS calculated for $C_{20}H_{19}N_2O_6S^+$: 415.0958; found 415.0949.

11. Synthesis of 4-(5-((3-(2-morpholino-2-oxoethyl)-2,,4-dioxothizolidin-5-ylidene)methyl)furan-2-yl)phenyl) dihydrogen phosphate 4c

5-((5-(4-hydroxyphenyl)furan-2-yl)methylene)-3-(2-morpholino-2-oxoethyl)thiazolidine-2,4-dione **4b** (0.9 mmol, 1 eq.) was dissolved in anhydrous DCM (15 mL) under a N_2 atmosphere. Et_3N (2.7 mmol, 3 eq.) was added at room temperature, and the mixture was cooled to 0 °C, followed by the addition of diethyl chlorophosphate (1.1 mmol, 1.2 eq.). The reaction mixture was stirred at 0 °C for 5 hours, then

the solvent was evaporated under reduced pressure. The crude residue was purified by automated flash column chromatography eluting with *n*-hexane:EtOAc 70:30 v/v increasing to *n*-hexane:EtOAc 0:100 v/v in 15 CV, affording intermediate diethyl (4-(5-((3-(2-morpholino-2-oxoethyl)-2,4-dioxothizolidin-5-ylidene)methyl)furan-2-yl)phenyl) phosphate as a red solid in 89% yield. ^{1}H-NMR (DMSO-d$_6$), δ: 3.32 (s, 6H), 3.42-3.44 (m, 2H), 3.53-3.58 (m, 4H), 3.62-3.64 (m, 2H), 4.15-4.21 (m, 4H), 4.60 (s, 2H), 7.28-7.31 (m, 2H), 7.37-7.39 (m, 2H), 7.81 (s, 1H), 7.87-7.89 (m, 2H). ^{13}C-NMR (DMSO-d$_6$), δ: 16.3, 16.4, 42.4, 42.9, 45.0, 64.9, 65.0, 66.4, 117.7, 119.8, 121.4 (d, J_P= 4.9 Hz), 122.5, 126.2, 126.6, 149.3, 151.2 (d, J_P= 6.8 Hz), 157.0, 164.0, 165.5, 168.3. ^{31}P-NMR (DMSO-d$_6$), δ: -6.55.

Diethyl (4-(5-((3-(2-morpholino-2-oxoethyl)-2,4-dioxothizolidin-5-ylidene)methyl)furan-2-yl)phenyl) phosphate (0.12 mmol, 1 eq.) was dissolved in anhydrous DCM (5 mL) under a N_2 atmosphere, and the mixture was cooled to 0 °C. TMSBr (0.6 mmol, 5 eq.) was added, and the reaction mixture was stirred at room temperature overnight. After this time, further TMSBr (1.2 mmol, 10 eq.) was added at 0 °C, and the reaction mixture was stirred at room temperature for further 8 hours. MeOH (2 mL) was then added at 0 °C, and the reaction mixture was stirred at room temperature for 4 hours. The solvents were evaporated at reduced pressure, and the crude residue was purified by reverse-phase automated flash column chromatography eluting with H_2O:MeCN:FA 99.9:0:0.1 v/v increasing to H_2O:MeCN:FA 0:99.9:0.1 v/v in 12 CV, affording the title compound as a yellow solid in 49% yield. ^{1}H-NMR (DMSO-d$_6$), δ: 3.07-3.08 (m, 2H), 3.43-3.45 (m, 2H), 3.57-3.59 (m, 2H), 3.64-3.65 (m, 2H), 4.61 (s, 2H), 7.20 (d, J= 3.7 Hz, 1H), 7.29 (d, J= 3.7 Hz, 1H), 7.31 (d, J= 8.5 Hz, 2H), 7.78 (s, 1H), 7.79-7.80 (m, 2H). ^{13}C-NMR (DMSO-d$_6$), δ: 42.7, 42.9, 45.1, 49.2, 65.5, 109.5, 117.2, 117.9 (d, J_P= 0.9 Hz), 118.8, 119.9, 121.4, 122.8, 126.3, 149.0, 157.8, 164.1, 165.6, 168.5. ^{31}P-NMR (DMSO-d$_6$), δ: -5.89. UPLC-MS (Method C): t_R 1.51 min, MS [ESI, m/z]: 495.1 [M+H]$^+$. HRMS calculated for $C_{20}H_{20}N_2O_9PS^+$: 495.0622; found 495.0619.

12. Synthesis of 4-(5-((3-(2-morpholino-2-oxoethyl)-2,,4-dioxothizolidin-5-ylidene)methyl)furan-2-yl)phenyl) dihydrogen phosphate 5a

1-(4-Hydroxyphenyl)pyrazolidine-3,5-dione **62** (0.4 mmol, 1 eq.) and 4-(5-formylfuran-2-yl)-*N*-phenylbenzenesulfonamide **63** [1] (1 eq, 0.117 g, 0.357 mmol) were suspended in acetic acid (7 mL) and the reaction was stirred at 120°C for 3 hours. The mixture was then cooled to room temperature, quenched by the addition of deionized water (15 mL), and the precipitate formed was collected by filtration and washed thoroughly with water and *n*-hexane. The crude residue was purified by recrystallisation from DCM, affording the title compound as a brown solid in 72% yield. ^{1}H-NMR (DMSO-d$_6$), δ: 6.83-6.85 (m, 2H), 7.04-7.08 (m, 1H), 7.11-7.13 (m, 2H), 7.24-7.27 (m, 2H), 7.49-7.51 (m, 2H), 7.55-7.56 (m, 1H), 7.64-7.65 (m, 1H), 7.85-7.87 (m, 2H), 8.09-8.12 (m, 2H), 8.42 (bs, 1H), 9.57 (bs, 1H), 10.36 (bs, 1H). ^{13}C-NMR (DMSO-d$_6$), δ: 113.6, 113.7, 115.7, 115.8, 120.9, 121.0, 124.8, 124.9, 126.0, 128.0,

128.1, 129.7, 132.7, 137.9, 140.2, 150.8, 150.9, 157.6, 172.5, 176.6. UPLC-MS (Method C): t_R 1.74 min, MS [ESI, m/z]: 502.1 [M+H]$^+$. HRMS calculated for $C_{26}H_{20}N_3O_6S^+$: 502.1067; found 502.1071.

13. Synthesis of (2-(4-hydroxyphenyl)-3,5-dioxo-4-((5-(4-(N-phenylsulfamoyl)phenyl)furan-2-yl)methylene)pyrazolidin-1-yl)phosphonic acid 64

4-(5-((1-(4-Hydroxyphenyl)-3,5-dioxopyrazolidin-4-ylidene)methyl)furan-2-yl)-*N*-phenylbenzenesulfonamide **5b** (0.4 mmol, 1 eq.) was dissolved in anhydrous DCM (13 mL) under a N_2 atmosphere. The mixture was cooled to 0 °C, followed by the dropwise addition of a solution of diethyl chlorophosphate (0.4 mmol, 1 eq.) in anhydrous DCM (6.5 mL). The reaction mixture was stirred at 0 °C for 3 hours, then Et_3N (0.4 mmol, 1 eq.) was added dropwise at 0 °C. The resulting mixture was stirred at 0 °C overnight. The solvent was evaporated under reduced pressure, and the crude residue was purified by automated flash column chromatography eluting with DCM:MeOH 100:0 v/v increasing to DCM:MeOH 95:5 v/v in 15 CV, affording intermediate 4-(3,5-dioxo-4-((5-(4-*N*-phenylsulfamoyl)phenyl)furan-2-yl)methylene)pyrazolidin-1-yl)phenyl diethyl phosphate in a 2:1 ratio with the structural isomer diethyl (*E*)-((4-(5-((1-(4-hydroxyphenyl)-3,5-dioxopyrazolidin-4-ylidene)methyl)furan-2-yl)phenyl)sulfonyl)(phenyl)phosphoramidate. The 2:1 mixture of isomer was obtained as a brown solid in 66% yield. ^{1}H-NMR (DMSO-d_6), δ: *main species*: 1.35 (dt, J_1= 7.0 Hz, J_2= 1.1 Hz, 6H), 4.30-4.36 (m, 4H), 6.82-6.85 (m, 2H), 7.02-7.06 (m, 1H), 7.09-7.12 (m, 2H), 7.22-7.26 (m, 2H), 7.54-7.58 (m, 2H), 7.62-7.63 (m, 1H), 7.64 (s, 1H), 7.85-7.88 (m, 2H), 8.12-8.14 (m, 2H), 8.63 (bs, 1H), 9.52 (bs, 1H), 10.36 (bs, 1H); *minor species*: 1.16 (dt, J_1= 7.0 Hz, J_2= 1.0 Hz, 6H), 4.22-4.13 (m, 4H), 6.82-6.85 (m, 2H), 7.02-7.06 (m, 1H), 7.09-7.12 (m, 2H), 7.22-7.26 (m, 2H), 7.54-7.58 (m, 2H), 7.58-7.59 (m, 1H), 7.66 (s, 1H), 7.83-7.85 (m, 2H), 8.16-8.19 (m, 2H), 8.63 (bs, 1H), 9.50 (bs, 1H), 10.36 (bs, 1H). ^{13}C-NMR (DMSO-d_6), δ: *main and minor species*: 15.6 (d, J_P= 6.4 Hz), 15.8 (d, J_P= 6.6 Hz), 65.0 (d, J_P= 5.8 Hz), 65.3 (d, J_P= 6.2 Hz), 112.7 (d, J_P= 5.0 Hz), 113.4, 113.7, 115.2, 115.3, 120.3, 120.4, 120.9, 124.3, 124.4, 125.7, 126.0, 127.3, 127.6, 128.7, 128.8, 129.1, 129.2, 129.5, 129.6, 131.7, 131.8, 137.3 (d, J_P= 1.1 Hz), 140.0, 149.8, 150.3, 150.4 (d, J_P= 7.8 Hz), 154.6, 154.8, 158.2, 158.5, 159.0, 160.9. ^{31}P-NMR (DMSO-d_6), δ: *main species*: -7.91; *minor species*: -7.51.

The 2:1 isomeric mixture of diethyl phosphates (0.2 mmol, 1 eq.) was dissolved in anhydrous DCM (7 mL) and anhydrous DMF (0.5 mL) under a N_2 atmosphere, and the mixture was cooled to 0 °C. TMSBr (2 mmol, 10 eq.) was added dropwise, and the reaction mixture was stirred at room temperature for 2.5 hours. After this time, further TMSBr (1 mmol, 5 eq.) was added at 0 °C, and the reaction mixture was stirred at room temperature overnight. MeOH (3 mL) was then added at 0 °C, and the reaction mixture was stirred at room temperature for 1.5 hours. The solvents were evaporated at reduced pressure, and the crude residue was purified by automated flash column chromatography eluting with DCM:MeOH:AcOH 100:0:0 v/v increasing to DCM:MeOH:AcOH 75:22:3 v/v in 12 CV, affording the

title compound in a 2:1 ration with structural isomer ((4-(5-((1-(4-hydroxyphenyl)-3,5-dioxopyrazolidin-4-ylidene)methyl)furan-2-yl)phenyl)sulfonyl)(phenyl)phosphoramidic acid **65**. The 2:1 mixture of isomers was obtained as a brown solid in 58% yield. ^{1}H-NMR (DMSO-d_6), δ: *main species*: 7.03-7.06 (m, 1H), 7.10-7.12 (m, 2H), 7.22-7.26 (m, 4H), 7.51-7.58 (m, 1H), 7.65-7.71 (m, 3H), 7.78-7.86 (m, 3H), 8.07-8.11 (m, 2H), 10.36 (bs, 1H); *minor species*: 7.03-7.06 (m, 1H), 7.10-7.12 (m, 2H), 7.22-7.26 (m, 4H), 7.51-7.58 (m, 2H), 7.78-7.86 (m, 1H), 8.07-8.11 (m, 1H), 8.14-8.27 (m, 2H), 8.37-8.39 (m, 2H), 8.48-8.49 (m, 1H), 8.60 (bs, 1H), 10.35 (bs, 1H). ^{13}C-NMR (DMSO-d_6), δ: *main and minor species*:113.1, 114.8, 115.0, 119.6, 120.0, 120.2, 120.3, 120.4, 124.3 (d, J_P= 2.3 Hz), 125.4, 127.4 (d, J_P= 4.0 Hz), 129.0, 131.9, 137.2, 139.6, 150.2 (d, J_P= 5.4 Hz), 157.1 (d, J_P= 10.2 Hz). ^{31}P-NMR (DMSO-d_6), δ: *main species*: -6.22; *minor species*: -1.16. UPLC-MS (Method C): t_R 1.58 min, MS [ESI, m/z]: 582.1 [M+H]$^+$.

Figure S1. Dose response curves for compounds 4b and 5a.

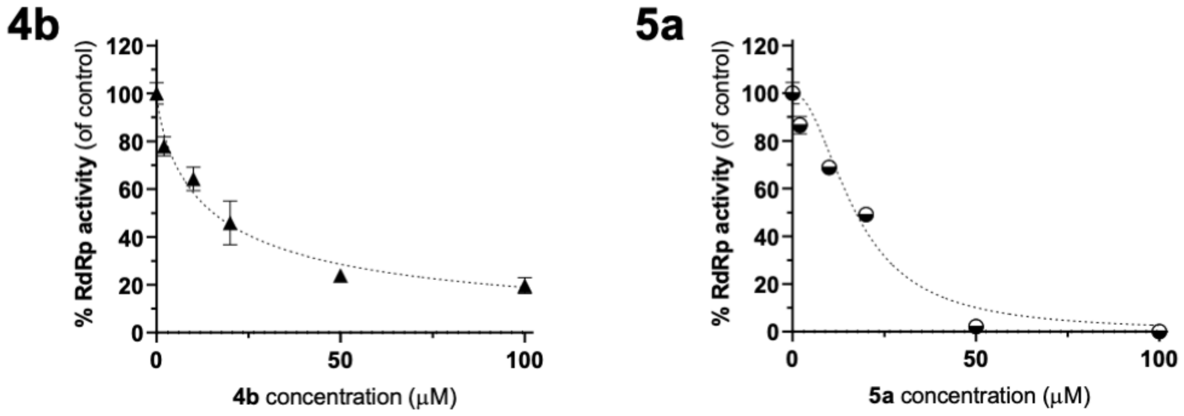

Figure S2. Full gels for the gel-shift confirmatory assay. A gel-shift assay was used to confirm norovirus RdRp inhibitory activity. PE44-NoV RNA templates (32 nucleotides, 5′ CCCCCCCCCCCCGGCCGGCCGUAAGGCCGGCC -3′) were extended (44 nucleotides) by RdRp in the absence of any test compounds (0.5% DMSO [vol/vol] negative control) or with test compounds at a fixed concentration of 100 μM. PPNDS (100 μM) and **5** were used as positive controls to demonstrate complete inhibition, and no RdRp was used as a negative control. Compounds labelled as a red rectangle were not included in this article.

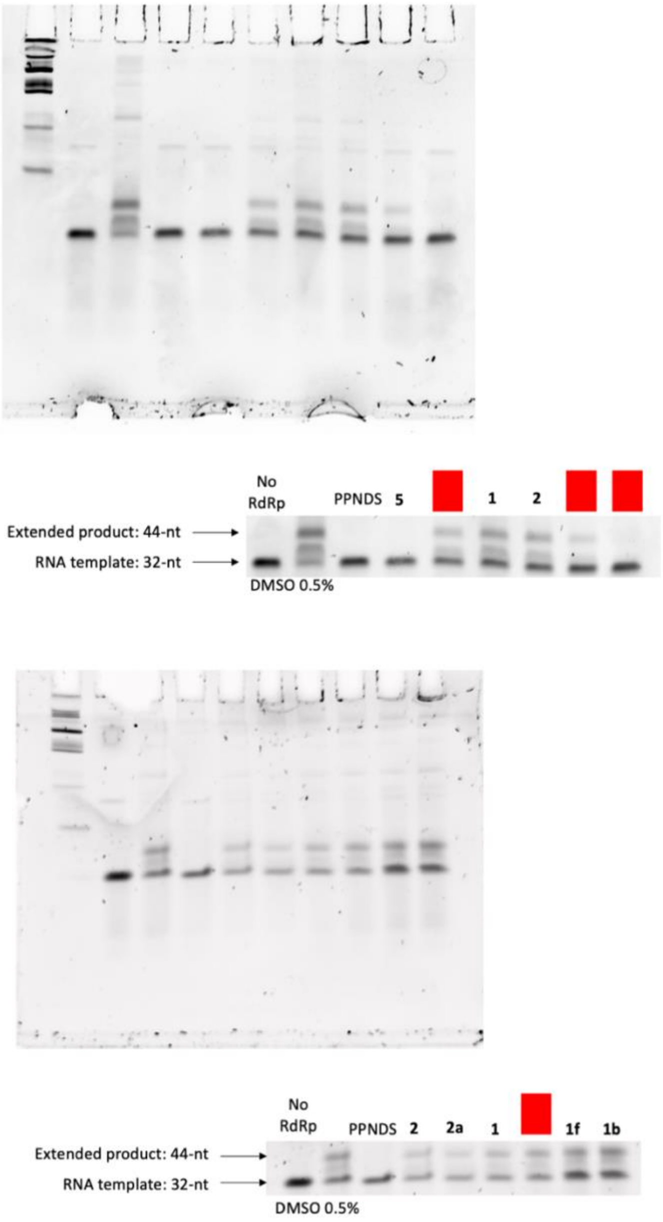

Figure S2. Full gels for the gel-shift confirmatory assay. Continued from previous page.

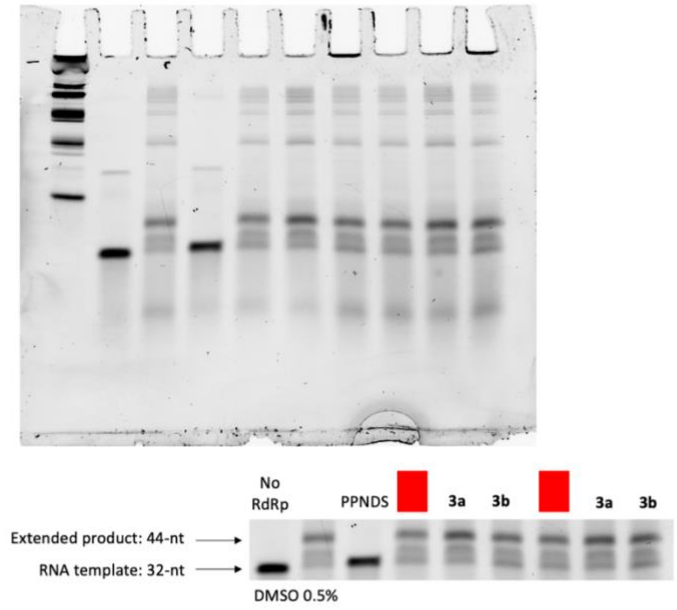

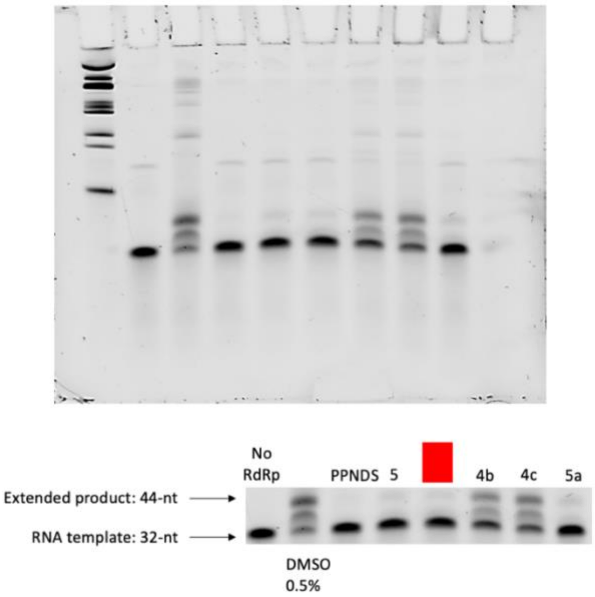

Table S1. Antiviral and cytotoxicity data for the test compounds

Comp.	Structure	MNV EC_{50}[A] (μM)	MNV CC_{50}[A] (μM)
**1**	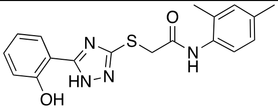	>100	42.1±16.9
**1a**	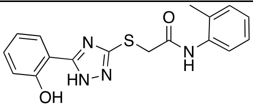	>100	56.1±18.8
**1b**	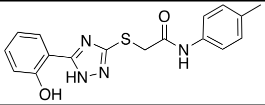	>100	42.9±26.8
**1c**	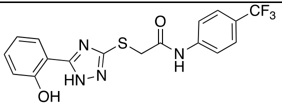	>100	18.29±15.01
**1d**	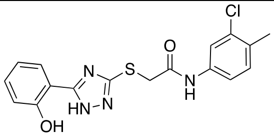	>100	15.99±12.31
**1f**	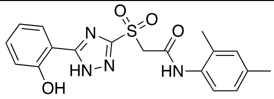	>100	>100
**2**	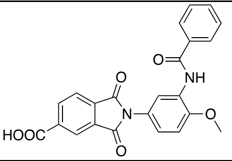	>100	>100
**2a**	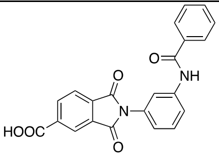	>100	>100
**2b**	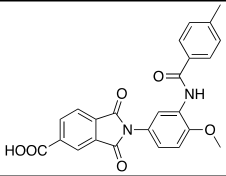	>100	>100
**2c**	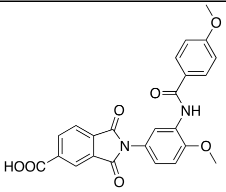	>100	>100
**2d**	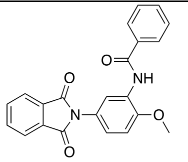	>100	>100
**2e**	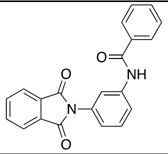	>100	>100

**2f**	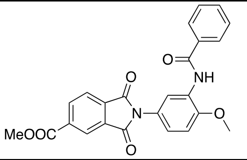	>100	>100
**2g**	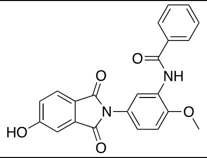	>100	>100
**3**	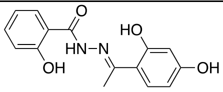	>100	50.58
**3a**	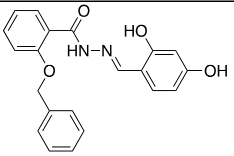	>100	>100
**3b**	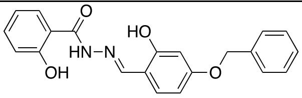	97.7±3.2	50.8±69.4
**4**	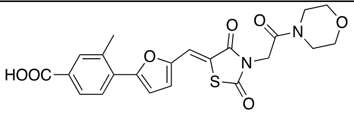	>100	>100
**4b**	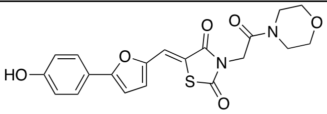	>100	17.6±4.6
**5**	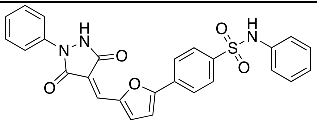	>100	10.86±0.78
**5a**	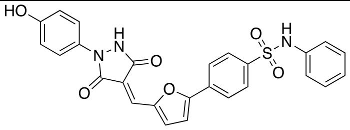	>100	5.2±1.4
**2-CMC**	Positive control	16.2±5.9	36.5±7.7

[A] The mean values ± standard deviations are shown from at least three independent experiments.

Figure S3. 2D interaction diagrams for the docking results.

1

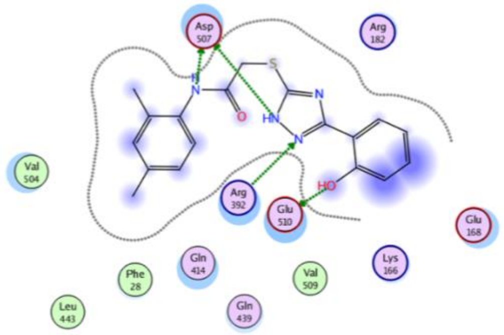

1e

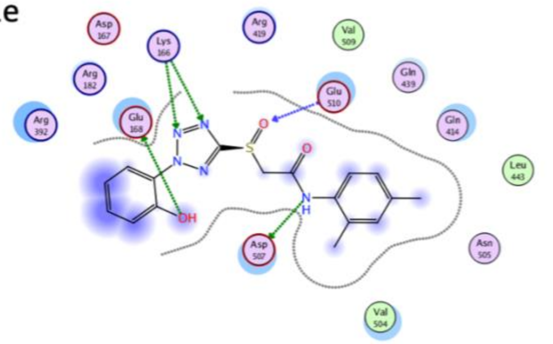

1f

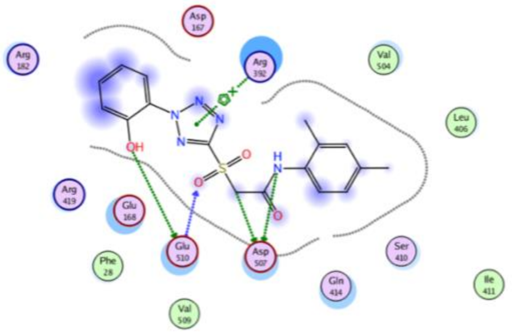

2

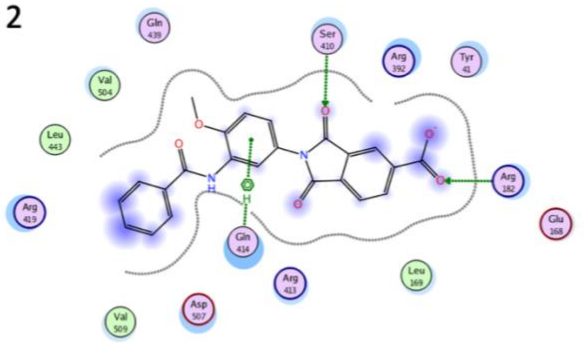

2g

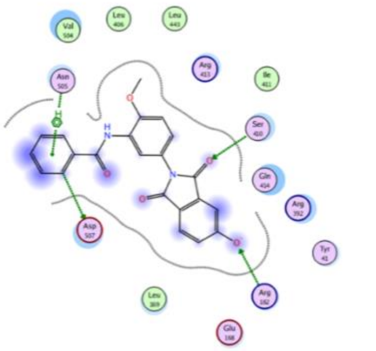

2h

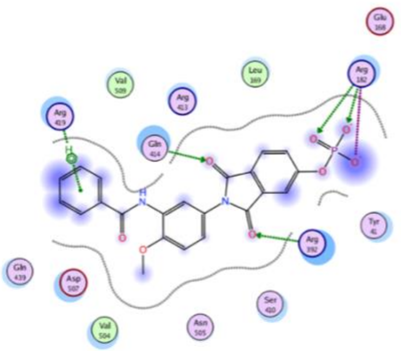

3

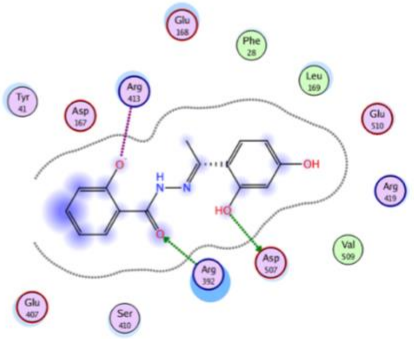

3a

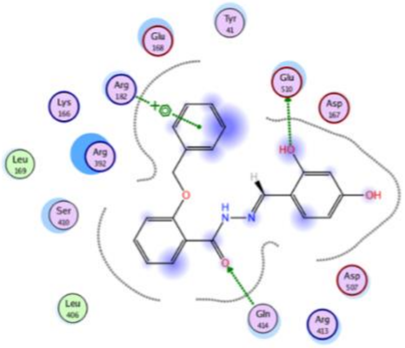

Figure S3. 2D interaction diagrams for the docking results. Continued from previous page.

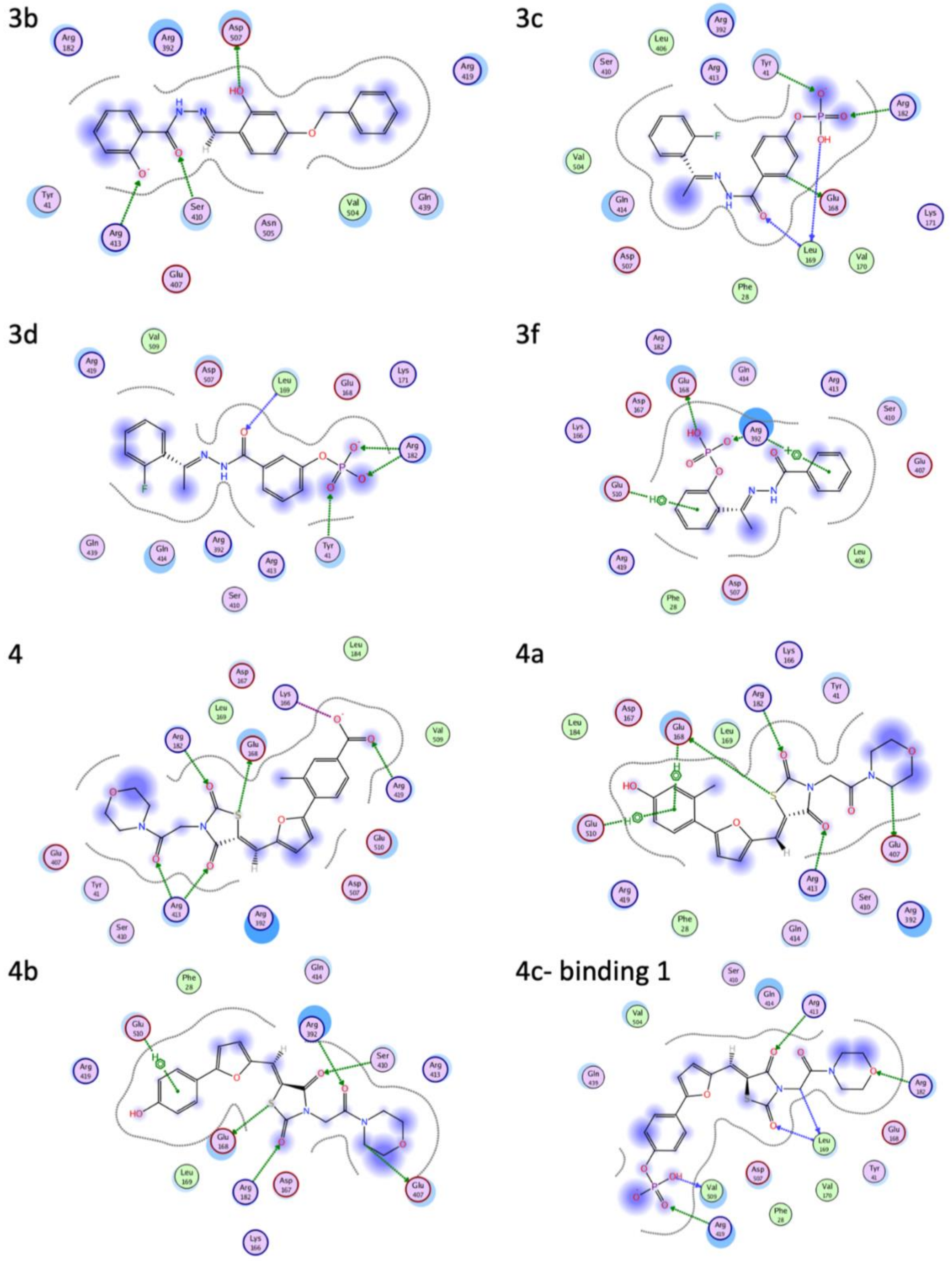

Figure S3. 2D interaction diagrams for the docking results. Continued from previous page.

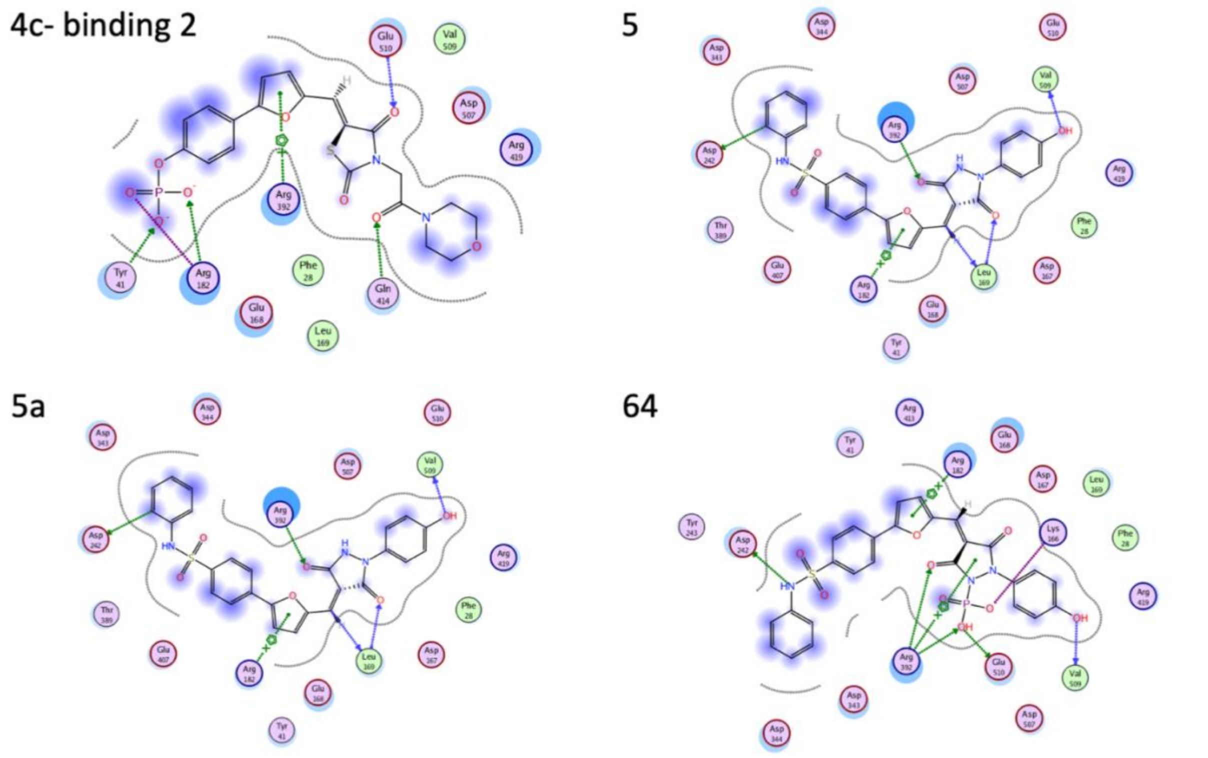

References

1. Ferla, S.; Netzler, N.E.; Ferla, S.; Veronese, S.; Tuipulotu, D.E.; Guccione, S.; Brancale, A.; White, P.A.; Bassetto, M. In silico screening for human norovirus antivirals reveals a novel non-nucleoside inhibitor of the viral polymerase. *Scientific Reports* **2018**, 8, 4129.
